# *De novo* assembly of Sockeye salmon kidney transcriptomes reveal a limited early response to piscine reovirus with or without infectious hematopoietic necrosis virus superinfection

**DOI:** 10.1186/s12864-016-3196-y

**Published:** 2016-11-02

**Authors:** Mark P. Polinski, Julia C. Bradshaw, Sabrina M. Inkpen, Jon Richard, Camilla Fritsvold, Trygve T. Poppe, Matthew L. Rise, Kyle A. Garver, Stewart C. Johnson

**Affiliations:** 1 Fisheries and Oceans Canada, Pacific Biological Station, 3190 Hammond Bay Rd, Nanaimo, BC V9T6N7 Canada; 2 Department of Ocean Sciences, Memorial University, St. John’s, NF A1C5S7 Canada; 3 Department of Pathology, Norwegian Veterinary Institute, Oslo, NO-0106 Norway; 4 Department of Basic Sciences and Aquatic Medicine (Basam), Norwegian University of Life Sciences, P.O. Box 8146, Dep, N-0033 Oslo, Norway

**Keywords:** Sockeye salmon, Piscine reovirus, Infectious hematopoietic necrosis virus, RNA-seq, Transcriptome, *De novo* assembly

## Abstract

**Background:**

Piscine reovirus (PRV) has been associated with the serious disease known as Heart and Skeletal Muscle Inflammation (HSMI) in cultured Atlantic salmon *Salmo salar* in Norway. PRV is also prevalent in wild and farmed salmon without overt disease manifestations, suggesting multifactorial triggers or PRV variant-specific factors are required to initiate disease. In this study, we explore the head kidney transcriptome of Sockeye salmon *Oncorhynchus nerka* during early PRV infection to identify host responses in the absence of disease in hopes of elucidating mechanisms by which PRV may directly alter host functions and contribute to the development of a disease state. We further investigate the role of PRV as a coinfecting agent following superinfection with infectious hematopoietic necrosis virus (IHNV) – a highly pathogenic rhabdovirus endemic to the west coast of North America.

**Results:**

Challenge of Sockeye salmon with PRV resulted in high quantities of viral transcripts to become present in the blood and kidney of infected fish without manifestations of disease. *De novo* transcriptome assembly of over 2.3 billion paired RNA-seq reads from the head kidneys of 36 fish identified more than 320,000 putative unigenes, of which less than 20 were suggested to be differentially expressed in response to PRV at either 2 or 3 weeks post challenge by DESeq2 and edgeR analysis. Of these, only one, Ependymin, was confirmed to be differentially expressed by qPCR in an expanded sample set. In contrast, IHNV induced substantial transcriptional changes (differential expression of > 20,000 unigenes) which included transcripts involved in antiviral and inflammatory response pathways. Prior infection with PRV had no significant effect on host responses to superinfecting IHNV, nor did host responses initiated by IHNV exposure influence increasing PRV loads.

**Conclusions:**

PRV does not substantially alter the head kidney transcriptome of Sockeye salmon during early (2 to 3 week) infection and dissemination in a period of significant increasing viral load, nor does the presence of PRV change the host transcriptional response to an IHNV superinfection. Further, concurrent infections of PRV and IHNV do not appear to significantly influence the infectivity or severity of IHNV associated disease, or conversely, PRV load.

**Electronic supplementary material:**

The online version of this article (doi:10.1186/s12864-016-3196-y) contains supplementary material, which is available to authorized users.

## Background

Piscine orthoreovirus (PRV) is a non-enveloped, double stranded RNA virus which is a member of the family *Reoviridae* [1]. Its genome consists of 10 nucleic acid segments L1, L2, L3, M1, M2, M3, S1, S2, S3, and S4 that encode for the λ3, λ2/p11, λ1, μ2, μ1, μNS, σ3/p13, σ2/p8, σNS, and σ1 proteins, respectively [1–3]. PRV was first identified by pyrosequencing in 2010 as part of a Norwegian-based study to identify the causative agent of a recently emerged disease of cultured Atlantic salmon (*Salmo salar*) known as Heart and Skeletal Muscle Inflammation (HSMI) [1]. In that study, high levels of PRV transcripts were found in 28 of 29 diseased specimens, suggesting an association between PRV and HSMI. In Norway, this association has been strengthened by subsequent challenge studies and viral screenings from HSMI outbreaks which have confirmed that PRV is almost always detected during HSMI [4–8] and host antiviral responses are activated during this disease [8–11]. However, wild and farmed Atlantic salmon have also been shown to carry high loads of PRV in Norway without evidence of HSMI [1, 4, 12].

Shortly after its discovery, PRV was similarly identified by PCR in farmed Atlantic salmon on the west coast of North America (Marty and Bidulka, unpublished data). Subsequent studies have confirmed the presence of PRV in farmed Atlantic salmon in British Columbia [3, 13, 14], and also identified PRV in wild salmonids in both BC and US (Washington and Alaska) waters [3, 14, 15]. Interestingly, although infection with PRV is common in farmed Atlantic salmon in BC, disease manifestations impacting fish performance as observed in Norway has never been reported (reviewed in [16]). Using injection and cohabitation challenge trials, we recently examined the infectivity and pathogenicity of PRV from the west coast of North America to Atlantic and Sockeye salmon (*Oncorhynchus nerka*) and whether infection would result in HSMI [17]. We demonstrated that PRV was capable of infecting, replicating and persisting within both host species over a 41 week period yet histopathological signs of HSMI were not observed. As with infections associated with HSMI, these non-pathogenic infections showed a similar blood cell tropism and comparable infection dynamics, suggesting that PRV is of low virulence to Atlantic and Sockeye salmon in western North America [17].

The pathogenic versus non-pathogenic outcome associated with PRV infection is puzzling, and suggests multifactorial requirements or PRV variant-specific factors may be required to initiate PRV associated disease. As contributing factors from the host, pathogen(s), or environment are unknown with regard to the development of HSMI, it is important to systematically evaluate PRV pathogenesis prior to or in the absence of the possibly obfuscating physiological changes that may occur as a direct result of this inflammatory pathological state.

In the present study we take advantage of the apparent non-pathogenic relationship which occurs between PRV and Sockeye salmon in western North America to identify host-specific transcriptional responses during early systemic infection and increasing PRV load. We use next-generation RNA-sequencing (RNA-seq) technologies in conjunction with contemporary bioinformatics tools to describe and compare head kidney transcriptomes of PRV infected and non-infected Sockeye salmon. Guided by RNA-seq differential expression analyses, we further explore the significance of selected genes in expanded biological, tissue and temporal datasets with regard to PRV-specific host responses.

A second major objective of this study was to determine whether infection with PRV of no or low pathogenicity would affect how hosts respond to challenge with another RNA virus and if such co-infections could lead to PRV induced pathology; or, conversely, afford the host with a protective advantage against a superinfecting viral pathogen. As PRV has been shown to establish long-term infections in its hosts [17] and is common in natural systems along the western cost of Canada [14], it is inevitable that such co-infections will (or do) occur. One pathogen that is sympatric with PRV is infectious hematopoietic necrosis virus (IHNV); which occurs naturally in the waters of western North America and can cause acute disease (IHN) in nearly all salmonid species given appropriate viral, host, and environmental conditions. In British Columbia, IHNV predominately infects Sockeye salmon where much of the viral replication and associated pathology occurs in the kidney during acute infection [18] – an organ which also manifests high loads of PRV [7, 17] and has a significant proportion of blood associated cells to potentially indicate both systemic and organ-specific responses. We thus explore the consequences of a PRV infection on host cellular functions within the head kidney before and during superinfection with IHNV in hopes of better understanding such dynamics as may occur in natural environs.

## Methods

### Animal source and husbandry

Pitt River Sockeye salmon fry representing 140 families were obtained from Inch Creek Hatchery, British Columbia, Canada, and brought to the Pacific Biological Station (PBS) in Nanaimo, British Columbia, Canada. Fish were reared in 6 °C (±1 °C) dechlorinated freshwater under a natural photoperiod for 11 months at which time they were transitioned to seawater. Fish were reared in 11 °C (±1 °C) sand filtered UV-treated seawater for an additional 24 weeks prior to challenge. While in freshwater the fish were fed daily at 2.8 % body weight dry pellets (EWOS). Following transfer to seawater the feeding rate was reduced to 1 % body weight per day. Prior to their use in challenge trials a subsample of 20 fish was screened for the presence of PRV and IHNV using the methods described below.

### PRV intraperitoneal injection challenge

Sockeye salmon post-smolts were challenged with PRV by intraperitoneal (i.p.) injection following previously described methods [7, 17]. Inoculum containing PRV was prepared from pooled blood homogenates of 20 Atlantic salmon that had previously been shown to be infectious [17]. The mean load of reverse-transcribed PRV L1 transcripts in the PRV inoculum was approximately 7.1 × 10^5^ per 100 μL (Ct 20.3). A mock inoculum was prepared from pooled blood of 20 non-infected Atlantic salmon and confirmed to be PRV free by real-time quantitative polymerase chain reaction (qPCR) using methods described below. Nine hundred Sockeye salmon (approximately 40 g each) were i.p. injected with 100 μl of either PRV inoculum, mock inoculum, or L-15 media (300 fish per treatment) under 50 mg per L tricaine methanesulfonate (MS222) anaesthesia. Following injection, fish were assigned to four replicate tanks (75 fish per 360 L tank) per treatment group and held in 11 °C (±1 °C) sand filtered UV-treated seawater (32 ppt) in a 5–6 L per min flow-through system. Samples of blood and head kidney (approximately 50–100 μL and 50 mg, respectively) were collected from three fish from each tank (12 per treatment group) at 2, 5, 7 and 14 days post challenge (dpc). Clinical signs of disease, feeding performance, morbidity, water temperature, oxygen saturation and salinity were recorded daily.

### IHNV waterborne exposure

Fourteen days following challenge with PRV, half of the remaining Sockeye salmon (two tanks from each PRV treatment group) were exposed to an infectious dose of IHNV (genogroup U; isolate BC93-057) via waterborne exposure following previous methods [19]. Virus was propagated on *Epithelioma papulosum cyprini* (EPC) cells and brought to a final titre of 1 × 10^8^ plaque forming units (PFU) per mL in Hank’s Balanced Salt Solution (HBSS). Water flow was stopped and tank volume reduced to 100 L. Virus was added to a final concentration of 10^3^ PFU per mL and fish were held in the virus solution with aeration for one hour after which seawater flow was resumed and the tanks refilled to 360 L capacity. The remaining population (two tanks from each PRV treatment group) were mock challenged using HBSS under the same experimental conditions.

**Fig. 1:**
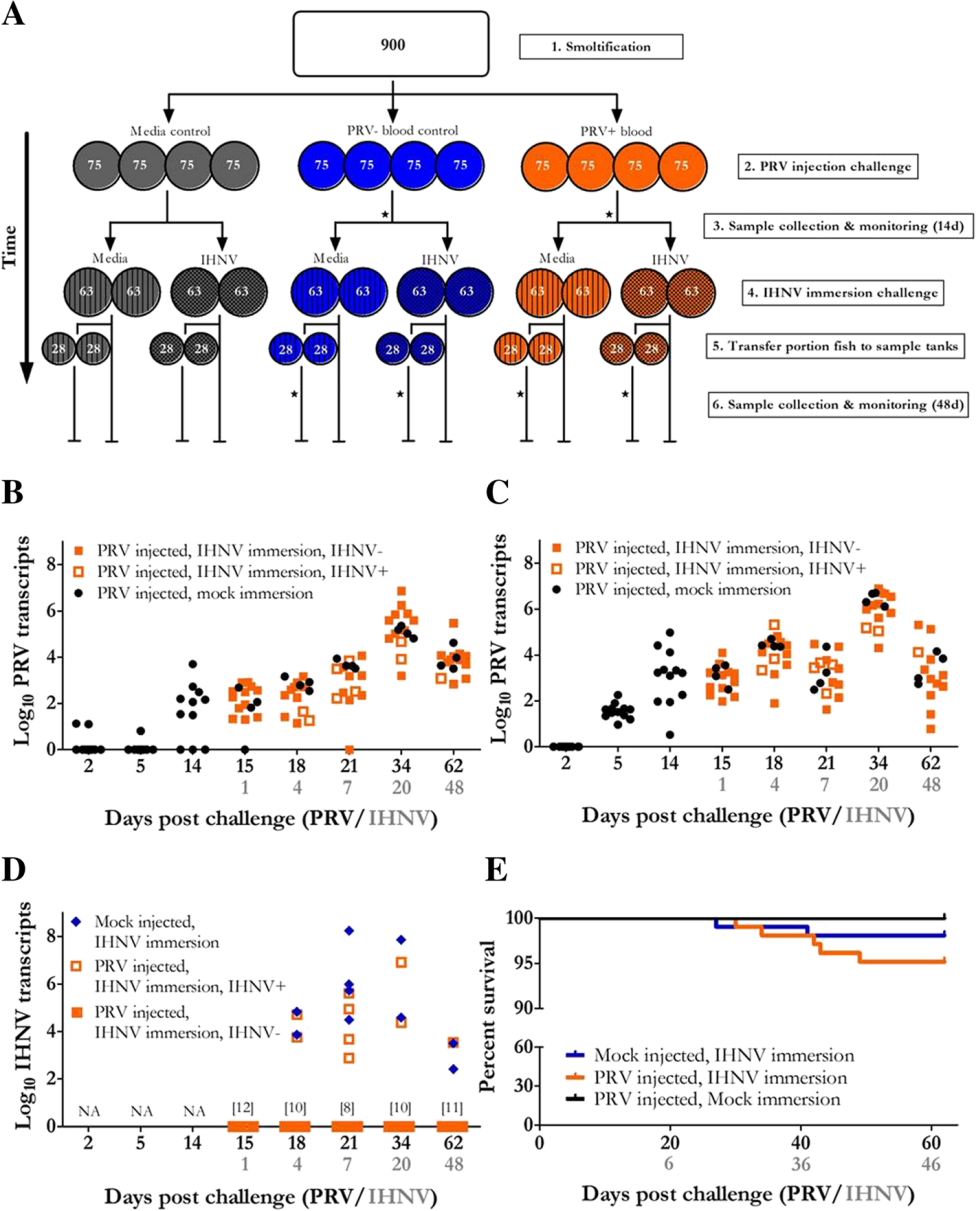
PRV and IHNV challenge of Sockeye salmon. **a** A schematic diagram presents the experimental design of this study. Circles represent experimental tanks and roman numerals indicate the number of fish initially added to each respective tank. 1.) 900 Sockeye salmon representing 140 families were acclimated to 12 °C seawater. 2.) Fish were equally divided into three groups and administered 100 μL injections of either: media control (*grey*), media/blood homogenate control (*blue*), or media/blood homogenate containing PRV (*orange*). 3.) Tissue and blood samples were collected from three fish in each tank at 2, 5, 7, and 14 days post challenge. 4.) Immediately following 14d sampling, two tanks in each PRV challenge group were administered a 1 h immersion of either: media control (*vertical lines*) or media containing IHNV (*checkerboard*). 5.) Four hours following immersion, 28 fish from each tank were moved to a smaller corresponding tank. 6.) Tissue and blood samples were collected from four fish in each of the smaller tanks at 1, 3, 7, 21 and 34 days post immersion. Fish remaining in the larger tanks (*n* = 35/tank) were monitored for morbidity without sampling disturbance. (★) indicates the populations and relative timing for samples collected that were used in RNA-seq transcriptome profiling. **b** Data presents PRV L1 transcripts per μg total extracted RNA for head kidney and (**c**) blood samples collected from fish exposed to PRV by intraperitoneal injection. Samples from fish subsequently exposed to IHNV by immersion that did not develop detectable IHNV kidney infections (IHNV-) are also distinguished from samples with detectable IHN virus (IHNV+). **d** Presents IHNV N transcripts per μg total RNA for head kidney samples where IHNV was detectable by qPCR (*n* = 19 infected of 120 challenged). Samples from fish previously exposed and harboring PRV L1 transcripts are distinguished from mock injected controls. The number of PRV challenged IHNV exposed samples for which IHNV was not detected by qPCR is provided in brackets. **e** Kaplan-Meier survival curves of fish exposed to PRV, IHNV, or PRV and IHNV presented in 62 day time course in reference to PRV challenge (*n* = 105 per treatment group). In all instances, relative days post challenge to IHNV superinfection is also provided

Four hours after the waterborne exposure, 28 of the 63 fish in each replicated treatment tank were removed and placed into corresponding 50 L circular tanks supplied with 1–2 L per min of 11 ° C filtered UV-treated seawater. Samples of blood and head kidney were collected from a portion of these fish (*n* = 4 fish per tank; 12 fish per treatment) at 1, 3, 7, 21 and 34 days post IHNV challenge. This corresponded to 15, 18, 21, 34 and 62 dpc with PRV, respectively. Remaining fish (*n* = 35) held in each larger 360 L tank were monitored for 48 days for clinical signs of disease and morbidity in the absence of sampling disturbance (Fig. 1a).

In addition to the samples described above, samples of head kidney and skeletal muscle from all treatment groups were taken for histopathology at 21 and 62 dpc PRV (7 and 48 dpc IHNV). Tissues were fixed in 10 % neutral buffered formalin for 24 h and then transferred to 70 % ethanol prior to paraffin embedding. The paraffin embedded samples were cut and stained with hematoxylin and eosin (H & E) [20] before histopathological evaluation by light microscopy.

### Viral detection and quantification

Blood and head kidney samples were tested for the presence of PRV by real-time qPCR as previously described [17]. Briefly, total RNA was extracted in TRIzol Reagent (Life Technologies) as per manufacturer’s instructions using 5 mm steel beads and TissueLyser II (Qiagen) which operated for 2 min at 25 Hz. A portion of eluted RNA (1.5 μg) was denatured for 1 min at 80 °C and reverse-transcribed using a High Capacity cDNA Reverse Transcription kit (Life Technologies) following the manufacturer’s instructions. Resulting cDNA was used directly as template for qPCR analysis in a StepOne-Plus real-time detection system (Applied Biosystems) using primers and TaqMan probe targeting the L1 fragment of the PRV genome (Additional file 1). Each reaction contained 400 nM primers (Eurofins Genomics) and 300 nM TaqMan probe (Life Technologies), 1X TaqMan Universal Master Mix and 2.5 μL cDNA template in a total of 25 μL. Cycling conditions included an initial incubation of 94 °C for 15 min followed by 45 cycles of 94 °C for 15 s, 54 °C for 30 s and 72 °C for 15 s.

The resulting cDNAs were also tested for the presence of IHNV by qPCR following methods previously described [21]. Briefly, primers and TaqMan probe targeting the nucleocapsid (N) gene of IHNV (Additional file 1) were combined in reactions containing 900 nM of each forward and reverse primers, 200 nM of IHNV N probe, 1X TaqMan Universal Master Mix and 2.5 μL cDNA template in a total of 25 μL. Cycling conditions were 50 °C for 2 min, 95 °C for 10 min, followed by 40 cycles of 95 °C for 15 s and 60 °C for 1 min. Fluorescence was measured at the end of each cycle on a StepOne-Plus real-time detection system.

All samples were assayed in duplicate for each virus and were considered positive if both technical replicates reported a Ct value < 40 cycles. Absolute quantification of reverse transcribed viral RNA was determined in each instance by serial dilution of a double-stranded DNA gBLOCK fragment (IDT Technologies) consisting of sequence targeted by the qPCR primers (probe) of each virus. A seven-step 10-fold dilution series of the gBLOCK fragment spanning a dynamic range of 10-10^7^ target copies per reaction was incorporated in duplicate into each run.

### RNA-seq

#### RNA isolation

Thirty-six samples of head kidney from two time points (14 and 21 dpc PRV) that consisted of eight experimental conditions (4–6 individuals per condition; Table 1) were selected for library construction and RNA-seq analysis. A portion (15 μg) of the total RNA extracted from these samples was purified using 2 U of DNase I (Life technologies) at 37 °C for 45 min followed by RNeasy MinElute Cleanup (Qiagen) as per manufacturer’s instructions. RNA quality was visualized on a 1 % bleach denaturing gel [22] and ensured to have a Bioanalyzer (Agilent) RNA Integrity Number (RIN) > 7.

**Table 1:** RNA-seq analysis of Sockeye salmon head kidney transcriptomes in response to PRV and IHNV challenge

Time point	PRV challenge & kidney status	IHNV challenge & kidney status	Library name	Accession	Raw reads	Surviving (%)
Group 1 [6 of 11]						
14 dpc PRV	Mock injected; PRV-	Not challenged	S86d14nHK	SRX1733826	71,073,165	65,851,201 (93)
			S87d14nHK	SRX1733827	67,245,066	61,789,431 (92)
			S88d14nHK	SRX1733828	70,681,709	65,014,139 (92)
			S89d14nHK	SRX1733829	71,447,579	65,600,555 (92)
			S91d14nHK	SRX1733830	64,978,759	61,093,604 (94)
			S92d14nHK	SRX1733831	66,681,946	60,949,173 (91)
Group 2 [6 of 12]						
14 dpc PRV	PRV injection; PRV+	Not challenged	S103d14pHK	SRX1733832	69,883,741	64,413,986 (92)
			S104d14pHK	SRX1733833	72,924,197	68,348,422 (94)
			S105d14pHK	SRX1733834	74,979,891	69,788,197 (93)
			S106d14pHK	SRX1733835	73,571,988	68,612,747 (93)
			S107d14pHK	SRX1733836	68,838,981	64,977,854 (94)
			S108d14pHK	SRX1733837	60,186,123	57,101,607 (95)
Group 3 [4 of 4]						
21 dpc PRV (7 dpc IHNV)	Mock injected; PRV-	Mock immersion; IHNV-	SM928d21nHK	SRX1733838	66,335,529	62,802,984 (95)
			SM929d21nHK	SRX1733839	67,164,004	62,826,291 (94)
			SM930d21nHK	SRX1733840	82,046,060	77,026,671 (94)
			SM931d21nHK	SRX1733841	70,537,334	66,494,922 (94)
Group 4 [4 of 8]						
21 dpc PRV (7 dpc IHNV)	Mock injected; PRV-	IHNV immersion; IHNV-	S194d21nHK	SRX1733842	70,095,033	66,369,408 (95)
			S195d21nHK	SRX1733843	67,157,595	63,977,824 (95)
			S197d21nHK	SRX1733844	71,994,014	68,166,660 (95)
			S203d21nHK	SRX1733845	50,101,150	47,036,872 (94)
Group 5 [4 of 4]						
21 dpc PRV (7 dpc IHNV)	Mock injected; PRV-	IHNV immersion; IHNV+	S193d21nHKIHN	SRX1733846	79,273,511	75,681,360 (95)
			S199d21nHKIHN	SRX1733847	76,263,972	72,360,305 (95)
			S200d21nHKIHN	SRX1733851	79,506,752	74,578,577 (94)
			S201d21nHKIHN	SRX1733862	78,032,333	74,005,961 (95)
Group 6 [4 of 4]						
21 dpc PRV (7 dpc IHNV)	PRV injection; PRV+	Mock immersion; IHNV-	SM932d21pHK	SRX1733881	67,630,420	64,227,495 (95)
			SM933d21pHK	SRX1733897	73,000,502	69,415,730 (95)
			SM934d21pHK	SRX1733920	83,809,559	79,117,126 (94)
			SM935d21pHK	SRX1733933	64,948,232	61,072,090 (94)
Group 7 [4 of 8]						
21 dpc PRV (7 dpc IHNV)	PRV injection; PRV+	IHNV immersion; IHNV-	S206d21pHK	SRX1733942	68,443,344	64,815,041 (95)
			S209d21pHK	SRX1733943	67,232,098	63,015,730 (94)
			S211d21pHK	SRX1733944	70,212,619	65,656,348 (94)
			S213d21pHK	SRX1733945	75,015,092	70,950,399 (95)
Group 8 [4 of 4]						
21 dpc PRV (7 dpc IHNV)	PRV injection; PRV+	IHNV immersion; IHNV+	S207d21pHKIHN	SRX1733946	73,284,580	69,850,430 (95)
			S210d21pHKIHN	SRX1733947	68,951,128	65,368,893 (95)
			S214d21pHKIHN	SRX1733948	68,150,625	64,986,061 (95)
			S216d21pHKIHN	SRX1733949	72,009,581	67,717,231 (94)

Libraries are organized into eight groups respective of unique experimental treatment, time points sampled and kidney infection status by both PRV and IHNV as assessed by qPCR. Associated libraries, NCBI SRA accession and sequencing coverage are provided in each instance. The numbers in brackets next to the group identifications indicate the number of samples screened by RNA-seq out of the total number of collected experimental samples available for that condition

#### Sequencing, *de novo* library construction and functional annotation

Library construction, sequencing services and bio-informatics support was provided by the McGill University and Génome Québec Innovation Centre, Montréal, Canada. A total of 36 libraries were generated from the experimental conditions as given in Table 1. RNA sequencing was performed using 12 lanes (3 libraries per lane) of an Illumina® HiSeq 2000 (Illumina Inc.) platform targeting polyadenylated mRNA prepared using a TruSeq V3 Kit with read lengths of 100 bp. Base calls were made using the Illumina CASAVA pipeline encoded in Phred 33. *De novo* transcriptome assembly was performed on resulting reads following the pipeline described by Haas *et al*. [23] based on the Trinity assembly software suite [24]. In brief, reads were trimmed using Trimmomatic software [25] from the 3’ end with a minimal Phred score of 30 and a minimum length of 32 bp. A normalized metric of reads was generated using Trinity normalization utility and surviving paired reads were assembled using the Trinity assembler [23]. Trinotate was used to identify putative coding transcripts and all putative transcripts were aligned against the UniProt 2013_11 protein database using the blastx program from the NCBI BLAST family. Annotation was assigned to each longest putative coding transcript derived from each de Bruijn graph component (unigene) based on highest blastx score with an Expect value (E) cut-off < 1e-5. First and second level Gene Ontology (GO) terms were compared using WEGO [26] for annotated unigenes with highest sequence similarity to other chordates. Gene abundance estimation for all transcripts was calculated using the RNA-Seq by Expectation-Maximization (RSEM) method [27] within the Trinity software framework.

#### Identification of differentially expressed genes

RSEM quantities calculated during d*e novo* assembly were used to assess differential unigene expression separately in both edgeR [28] and DESeq2 [29] Bioconductor software packages. Expressed non-informative unigenes without at least 0.5 counts per million mapped reads in at least four libraries were removed prior to either analysis using edgeR software. Differential expression was assessed using edgeR and DESeq2 based on the previously described protocols of Anders et al. [30] and Love et al. [31], respectively, in concert with the user guide information provided for each package on the Bioconductor website. A complete set of R-language commands and the session information applied in this study are provided for each analysis (see Additional file 2). Unigenes were considered differentially expressed at a *p* value < 0.05 following a Benjamini and Hochberg false discovery rate (FDR) adjustment of 5 % (0.05). To ensure higher stringency for putative differential regulation, unigenes were only considered for subsequent comparison and qPCR validated if they were identified as significant by both edgeR and DESeq2. Resulting differentially expressed unigenes were compared between analyses using VENNY [32].

### Gene expression by real-time qPCR

Total RNA was extracted from samples of head kidney and blood as described above. For each sample, 1.5 μg of DNAse-treated column-purified total RNA was reverse transcribed using a High Capacity cDNA Reverse Transcription Kit (Life Technologies) without RNase inhibitor following manufacturer’s instructions except that the random primer mix was substituted with 50 μM Olido d(T)_16_. Early time point (2 to 14 dpc PRV) qPCR was performed using a ViiA7 Real-Time PCR system (Applied Biosystems). PCR amplification was carried out in triplicate reactions containing Power SYBR Green master mix (Applied Biosystems), 50 nM forward and reverse primers (Additional file 1), and 2 μL of cDNA template (diluted 1:10 in water) in nuclease-free water to a total volume of 13 μL. Cycling conditions consisted of 50 °C for 2 min then 95 °C for 10 min, followed by 40 cycles of 95 °C for 15 s and 60 °C for 1 min, with fluorescence measured at the end of each 60 °C step. A portion of pooled cDNA was used to produce a five-step fourfold dilution series for assessing amplification efficiency and linearity in each run as previously described [33].

All other real-time qPCR analyses were conducted on a StepOne-Plus real-time detection system using SYBR green chemistry. Each PCR reaction consisted of 2X SYBR mastermix (Life Technologies), forward and reverse primers (500 nM each; Additional file 1), and 2 μL cDNA template (diluted 1:5 in water) to a final volume of 20 μL. Samples were assayed in duplicate with a five-step, fourfold dilution series of pooled cDNA included in each run to calculate amplification efficiency and linearity. Cycling conditions consisted of an initial activation of DNA polymerase at 95 °C for 10 min, followed by 40 cycles of 5 s at 95 °C, 25 s at 60 °C, and 10 s at 72 °C. At the end of all gene expression cycling protocols, melt curve analyses were run to ensure amplification specificity.

Target gene expression was normalized to the two most stable of four putative reference genes: elongation factor-1 alpha (*EF1-α*), beta-actin (*β-Actin*), acidic ribosomal protein (*ARP*) and Dynein (*Dyn*) as determined by the consensus of geNorm [34], BestKeeper [35],and NormFinder [36] software. Relative quantities were calculated from the qPCR raw fluorescence data using the global fitting mechanistic model of Carr and Moore [37] within the *qpcR* R-statistical software package [38]. Corrected normalized relative quantities were then compared at each time point by t-test or one-way ANOVA and Tukey post hoc test where appropriate using Graphpad Prism 5.0 following Log_10_ transformation of the data.

## Results

### Sockeye salmon were susceptible to PRV by i.p. injection and developed substantial blood and head kidney infections

Both head kidney and blood of naïve Sockeye salmon became highly infected with PRV following i.p. injection (Fig. 1). All fish sampled had detectible systemic levels of PRV L1 transcripts at 5 dpc. Viral loads in both blood and head kidney increased to 34 dpc, and then declined slightly by the end of the challenge (62 dpc PRV) (Fig. 1b & c). Mean reverse-transcribed PRV L1 transcripts at 34 dpc in blood and head kidney reached 3.2 × 10^6^ and 7.7 × 10^5^ copies per μg total RNA, respectively. These levels corresponded roughly to 2.4 × 10^8^ mean PRV L1 reverse-transcribed copies recovered from the combined portions of blood (~33 μg RNA) and head kidney (~173 μg RNA) sampled. These levels demonstrate that substantial PRV transcription occurred following challenge as each fish was initially i.p. challenged with < 1 × 10^6^ L1 copies of PRV. Levels of PRV in head kidney tissue used in library construction ranged from 1.1 × 10^2^ to 5.1 × 10^3^ copies per μg total RNA at 14 dpc and from 3.3 × 10^3^ to 8.8 × 10^3^ copies per μg total RNA at 21 dpc.

In this study i.p. challenge with PRV resulted in no morbidity or other signs of clinical disease over a 62 day period. In general, the histopathological lesions were few, mild and unspecific. As no heart tissue was available for histopathological evaluation, it was not possible to evaluate if any of the PRV infected fish developed HSMI. However, kidney and heart samples collected at 21 and 62 dpc revealed very few and mild lesions. Two fish had sparse, focal myositis in red carinal muscle, one fish had degenerative changes in red mid line muscle and two fish had interfibrillar exudation in red and white muscle, respectively. Samples of the kidney were without pathological lesions. The changes observed in the skeletal muscle samples were very sparse, focal and almost evenly distributed between PRV challenged and unchallenged fish and do not resemble the muscle inflammation described for HSMI in Atlantic salmon [39]. A detailed report of the histopathological findings is provided as Additional file 3.

### Immersion challenge of IHNV resulted in limited numbers of Sockeye salmon developing kidney infections

Of those samples collected over the 48 day period post IHNV challenge only 19/120 (16 %) individuals tested positive by qPCR for IHNV in the head kidney (Fig. 1d). Prevalence of IHNV, as well as mean viral load, was highest at 7 dpc challenge with IHNV, corresponding to 33 % and 2.2 × 10^7^ IHNV N reverse-transcribed transcripts per μg total RNA, respectively. As seen for PRV, IHNV loads declined in the last samples taken at 48 dpc with IHNV.

Morbidity ranged from 0 to 11 % in infected tanks following IHNV exposure and occurred between 13 and 35 dpc (Fig. 1e). Although cumulative IHNV associated morbidity was highest in the PRV coinfection group (6.5 ± 1.7 % SE), this was not significantly different to the group which received mock PRV injections (1.9 ± 1.2 % SE) (Additional file 4). The most common histopathological finding in kidney samples was mild circulatory disturbances in the interstitial tissue (5 fish). In head kidney, one fish had hematopoietic tissue with multifocal pyknotic and karyorrhectic nuclei, and another fish had few, small foci of interstitial haemorrhages. Some fish had signs of degenerative or necrotic changes in the epithelium of a few tubuli. The finding of necrotic cells and pyknotic, karyorrhectic cells in haematopoietic tissue of the kidney in two IHNV challenged fish is probably a typical manifestation of IHN [40].

In muscle samples, the most abundant changes observed were degeneration of white and/or red muscle, sometimes combined with increased eosinophilia of the fibers, but without inflammatory responses to the changes. Also in this group, some muscle samples displayed what appeared to be exudation between red and/or white muscle, and a few specimens had a characteristic clear vacuolation of red muscle fibers. Few individuals had slight interfibrillar hypercellularity of red muscle, and one fish had a focal, very sparse myositis in red muscle. The vacuolization of red muscle fibers and interfibrillar muscular exudation could result from damaged vessels, not uncommon in IHN diseased fish, but the number and occurrence of this also in fish challenged with PRV only suggest that the observed changes are more likely to represent some kind of metabolic or physiologic imbalance.

### IHNV and PRV infection dynamics were unaltered during periods of coinfection

Neither exposure or acute superinfection with IHNV significantly altered the transcription of PRV L1 transcripts in Sockeye salmon head kidney or blood (Fig. 1b & c). In addition, the presence of PRV did not significantly affect IHNV loads (Fig. 1d) or the outcome of IHNV challenge with respect to prevalence of infection and morbidity (Fig. 1d & e). A complete sample inventory including viral load estimates and morbidity are provided in Additional file 4. The histopathologic findings in muscle samples from fish challenged with both PRV and IHNV are relatively similar to what was seen in fish challenged with IHNV only. In kidney, circulatory disturbances in the haematopoietic kidney tissue and degenerative changes to tubuli epithelium are the most common findings. A majority of the fish with degeneration of red and/or white muscle, the most abundant muscle change found (19 fish), were from fish challenged with IHNV, or IHNV and PRV, and these findings could be associated with one or both viral infections. However, the specimens for histopathological evaluation were in general of small size with less amount of relevant tissue than ideal for detecting lesions related to PRV or IHN, resulting in many samples with artefacts that could obscure the interpretation of potentially disease specific and other pathological changes.

### A head kidney reference transcriptome for Sockeye salmon was created from more than 2.3 billion RNA-seq reads

RNA-seq libraries were generated for 36 samples of Sockeye salmon head kidney from eight discrete treatment conditions (Table 1). Surviving paired reads greater than 32 bp following trimming ranged from 47 to 80 million per library which represented 91-95 % of the raw sequence reads (Table 1). All libraries have been deposited in the NCBI Sequence Read Archive, study SRP0740078. Individual SRA library accession numbers are provided in Table 1.

To support the *de novo* assembly of our RNA-seq data, we used the Trinity normalization utility in order to reduce memory requirements needed for assembly. Following normalization this resulted in 40,403,129 surviving paired reads from which the reference transcriptome was assembled. *De novo* assembly yielded a total of 501,829 unique putative transcripts (Additional file 5) with a total length of all transcripts of 456,755,195 (bp), an average transcript length of 901 (bp), a median transcript length 464 (bp) and a maximum transcript length of 17,772 (bp). These transcripts represent 326,410 unigenes; of which, approximately 15 % (47,429) were suggested as being protein-coding following Trinotate and UniProt bastx annotation analysis (Additional file 6). However, this is likely a conservative estimate, as blastx predictions using current or future protein databases which include more annotated genes from Sockeye or other closely related species may identify additional protein-coding genes missed during our current analyses. Further, we set a minimum open reading frame threshold of 100 bp during Transdecoder protein prediction analysis, thereby excluding unigenes which may only code for shorter peptides.

**Fig. 2:**
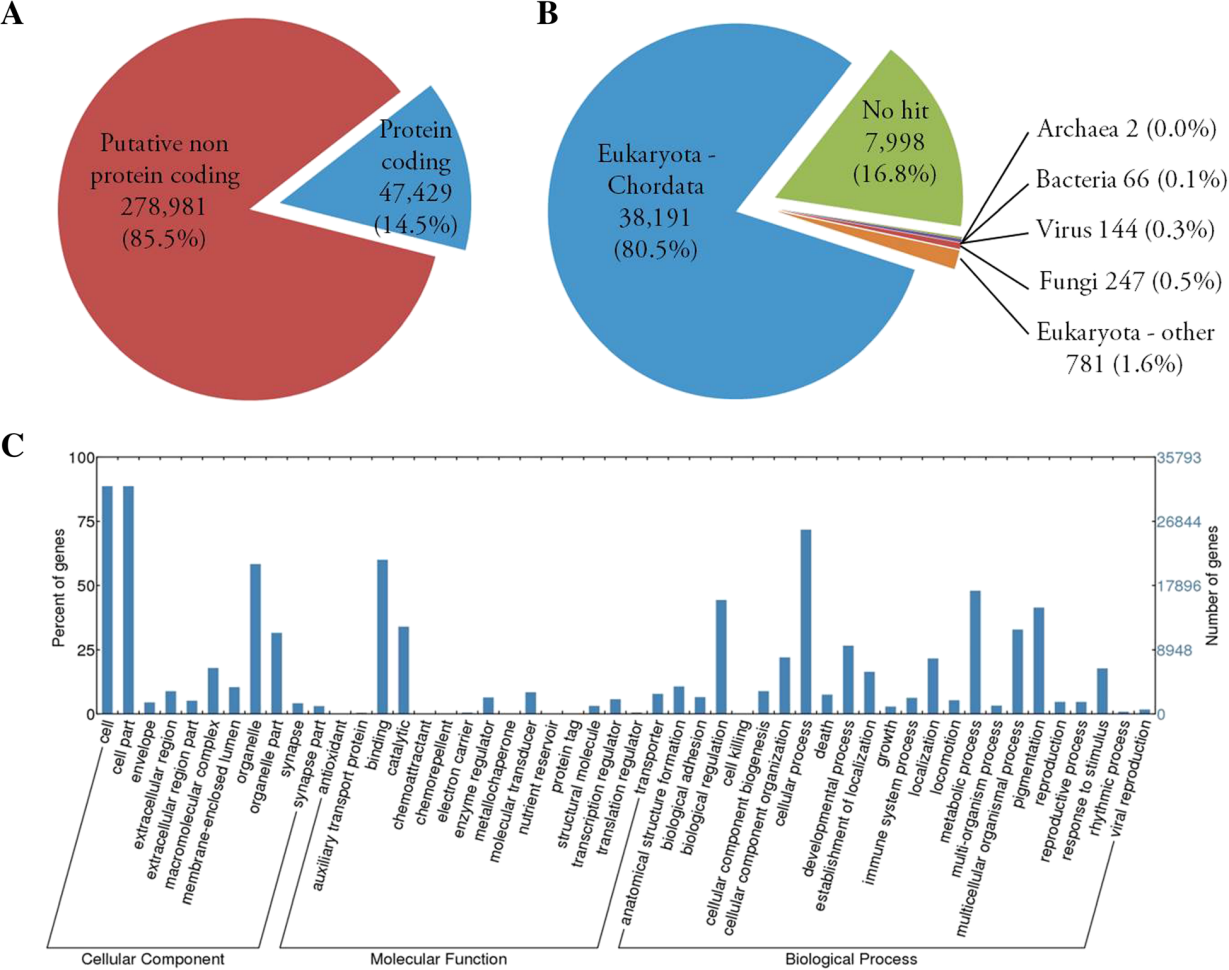
Trinity assembled component (unigene) summary for Sockeye salmon head kidney reference transcriptome. **a** Putative unigenes identified by Trinity in *de novo* assembly from 36 discrete head kidney libraries were determined to be either non- or protein-coding base on Transdecoder analysis and/or UniProt annotation. **b** All putative protein-coding unigenes are delineated by their best blastx hit in the UniProt 2013_11 database. **c** Unigenes with blast hits associated with Eukaryotic Chordates are categorized by first and second level gene ontology classifiers

Gene ontology classification of putative protein-coding unigenes which had high association to Eukaryotic Chordates (38,191) revealed 11, 16 and 23 functional groups classified as either cellular component, molecular function, or biological process, respectively (Fig. 2). Abundance estimates (RSEM counts) for all unigene associated transcripts identified in each RNA-seq library is provided in Additional file 7.

In addition to predictive chordate associated protein-coding genes, 1240 unigenes showed highest blastx similarity to non-chordate and presumably parasitic or commensal organisms. Specifically, 144 putative viral genes were identified. Although the majority (117) corresponded to the Retroviridae family (Additional file 6), seven IHNV associated genes were identified. These genes code for the G, N, M, P, and L (three variants) viral proteins and were present in eight of the IHNV challenged libraries: S193d21nHKIHN, S199d21nHKIHN, S200d21nHKIHN, S201d21nHKIHN, S213d21pHK, S207d21pHKIHN, S214d21pHKIHN and S216d21pHKIHN. This corresponded to seven of the eight libraries identified by qPCR to have N transcripts as well as one of the eight IHNV exposed libraries for which qPCR screening did not detect viral N transcripts (S213d21pHK). A low number of counts (<200 for all five transcripts) were observed in all libraries except S199d21nHKIHN, where counts ranged from 7651 to 53,792 for all five viral transcripts identified. In general, RNA-seq counts for IHNV followed the same pattern of estimated copies identified by qPCR. No PRV-specific transcripts were detected in any RNA-seq generated libraries; however, this was not unexpected, as reoviral transcripts have been shown to lack the 3’ polyadenylation sequence used during the RNA-seq cDNA synthesis in this study [41].

### Global expression of RNA-seq libraries showed responsiveness to IHNV but not PRV infection

Prior to assessing differential gene expression between treatments we examined overall similarity in global expression between transcriptome libraries independent of treatment condition. To reduce complex multidimensional RESM count data into two dimensions of variation for effective visualization, we used the clustering algorithms of multidimensional scaling (MDA) and principal component analysis (PCA) in edgeR or DESeq2, respectively.

**Fig. 3:**
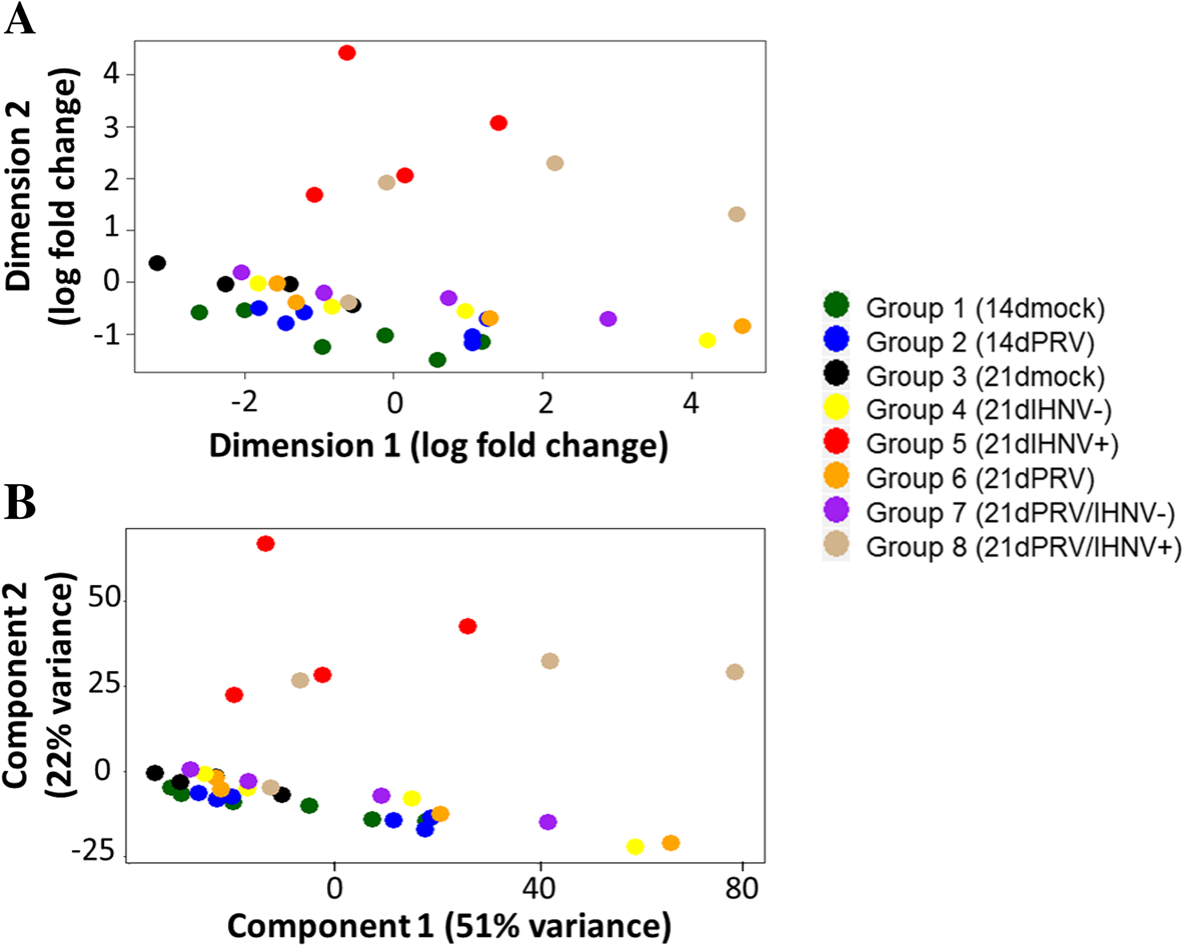
Similarities in global expression between RNA-seq libraries. Two-dimensional variance in global unigene expression of RNA-seq Sockeye salmon head-kidney libraries (*n* = 36) visualized by (**a**) multidimensional scaling analysis following edgeR normalization, or (**b**) principal component analysis following DESeq2 normalization and regularized-logarithm transformation. In each instance, collection relative to dpc PRV, exposure to PRV and/or IHNV, and IHNV kidney infection status (+/−) following challenge are indicated. For detailed experimental group conditions see Table 1

In both of these analysis, 4/4 individuals from Group 5 (21 day mock PRV injected, 7 day IHNV kidney infected) and 3/4 individuals from Group 8 (21 day PRV injected, 7 day IHNV kidney infected) orientated separately from individuals in the other groups which did not develop detectable IHNV kidney infections (Fig. 3a & b). Indeed, IHNV infections contributed strongly to the second highest source of variance (Y-axis) in both of these analyses. Although one individual from Group 8 was a non-responder (library S210d21pHKIHN), this library had the lowest quantity of detectable IHNV N transcripts in these groups as identified by qPCR (760 copies per μg total RNA) and was the only library in either Group 5 or 8 where IHNV transcripts were not identified by RNA-seq. Individuals in Groups 4 (21 day mock PRV injected, 7 day IHNV exposed) and 7 (21 day PRV injected, 7 day IHNV exposed), although challenged but without detectable IHNV kidney infections, had a slightly different ordination when compared to Groups 1, 2 and 3 and 6 which were never exposed to IHNV. Infection with PRV did not contribute to consistent library orientation and was uninformative in its overall effect on global expression by either MDA or PCA.

### PRV induced minor differential unigene expression changes relative to exposure or infection with IHNV in Sockeye head kidney

**Fig. 4:**
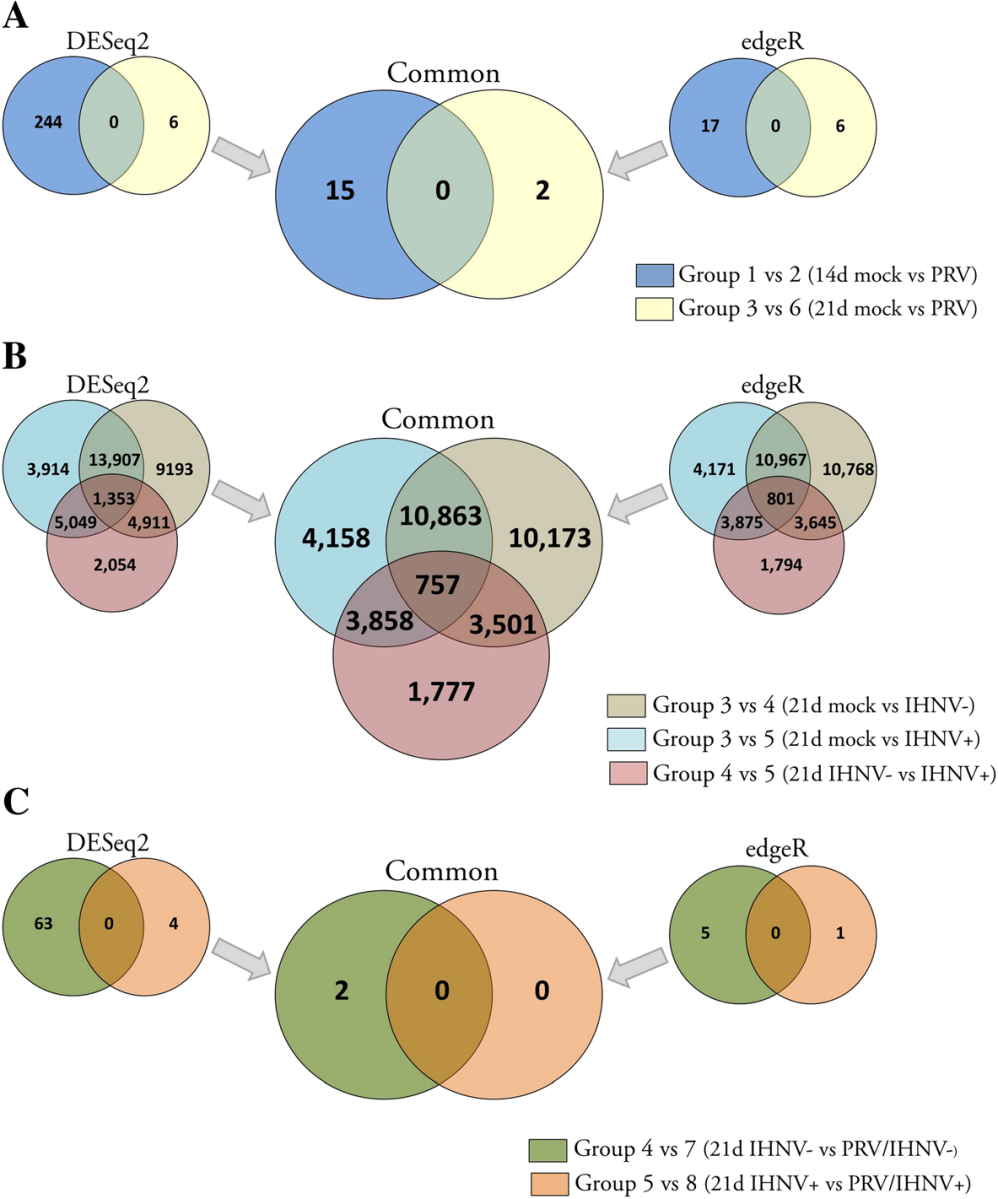
Differential unigenes expression in response to viral infections. Venn diagrams present the number of discrete and shared differentially expressed (both up and down regulated; 0.05 FDR-adjusted *p* < 0.05) unigenes identified by either DESeq2 (*left*), edgeR (*right*), or those common (*central*) to both analyses. Analyses were conducted using a reference transcriptome containing only genes with >0.5 cpm present in at least four libraries. Treatment-specific comparisons are presented as related to (**a**) PRV infection, (**b**) IHNV exposure and/or infection, or (**c**) those specific to PRV and IHNV co-infection. In each instance, collection relative to dpc PRV, challenge relative to PRV and/or IHNV, and IHNV kidney infection status (+/−) following exposure are indicated along with the experimental condition group number (see Table 1)

We compared the transcriptional response between mock and PRV challenged Sockeye salmon head kidneys at 14 (Group 1 vs. 2) and 21 (Group 3 vs. 6) dpc. At these time points PRV load in both the kidney and blood was increasing (Fig. 1b & c). Analyses conducted using DESeq2 or edgeR on RNA-seq count data revealed 250 and 23 differentially expressed unigenes (DEGs) with putative responsiveness to PRV, respectively (Fig. 4a). Higher numbers of DEGs were identified at 14 dpc when compared to 21 dpc in both analyses; however, none of the unigenes identified as differentially expressed at 14 dpc were shared with those identified at 21 dpc. With respect to the 250 DEGs identified by DESeq2, many appeared to be non-protein coding. Of the protein encoding genes, few appeared directly related to known antiviral pathways and normalized counts tended to be highly variable between individuals. When we considered only those genes which were shared between both DESeq2 and edgeR analyses 17 DEGs were identified. This represented 0.03 % out of the 60,421 pre-filtered unigene dataset used for each analysis. Of these, most (13) appeared to be non-protein coding (Table 2). The protein encoding genes had blastx annotations as Laminin subunit beta-1 and Nebulin at 14 dpc, and Ependymin and 18 s rRNA at 21 dpc.

**Table 2:** Putative PRV responsive unigenes

		edgeR		DESeq2		
Analysis	Unigene ID	Log_2_FC	*p* value	Log_2_FC	*p* value	blastx annotation
Group 1 vs 2 (14d mock vs PRV)						
	c197414_g1	0.9	0.0234	0.9	0.0002	
	c196659_g6	1.3	0.0456	1.2	0.0041	
	c194077_g5	1.7	0.0456	1.5	0.0112	
	c202007_g10	1.7	0.0456	1.5	0.0128	
	c191412_g1	1.7	0.0456	1.7	0.0106	
	c164000_g2	2.1	0.0260	1.8	0.0061	
	c191921_g6	8.0	0.0300	2.0	0.0480	Laminin subunit beta-1
	c189417_g6	2.4	0.0212	2.0	0.0106	
	c195179_g3	2.4	0.0006	2.1	0.0002	
	c192081_g1	2.4	0.0002	2.1	0.0001	Nebulin
	c190995_g6	2.8	0.0429	2.1	0.0141	
	c163909_g1	2.6	0.0104	2.2	0.0047	
	c191538_g2	3.1	0.0104	2.3	0.0051	
	c181963_g1	3.4	0.0212	2.5	0.0051	
	c197496_g5	4.0	0.0000	3.2	0.0000	
Group 3 vs 6 (21d mock vs PRV)						
	c162029_g1	3.0	0.0057	2.3	0.0030	Ependymin
	c198536_g3	3.9	0.0024	2.9	0.0003	18 s rRNA
Group 5 vs 8 (21d IHNV+ vs PRV/IHNV+)						
	c193589_g1	−2.8	0.0238	−2.3	0.0041	Matrix metalloproteinase-19
	c188458_g3	3.2	0.0238	2.5	0.0033	Histidine ammonia-lyase

Head kidney unigenes identified by both edgeR and DESeq2 as being differentially expressed in response to PRV. For each unigene, the Log_2_ fold change and 0.05 FDR adjusted *p*-value are presented as calculated by either edgeR or DESeq2 software packages. Best UniProt annotation (blastx E value < 1e-5) is provided where available

The low number of genes that were responsive to PRV infection was in strong contrast to the well-developed transcriptional responses seen following challenge with IHNV. At 7 dpc IHNV, there was as yet no morbidity as a result of IHNV; however, the highest prevalence and abundance of IHNV transcripts was observed at this time point in both PRV blood injected (4/12; mean 1.3 ± 0.9 SE × 10^5^ copies per μg RNA) and PRV- blood injected (4/12; mean 4.3 ± 4.2 SE × 10^7^ copies per μg RNA) challenged groups (Fig. 1). The number of putative DEGs that were common to both DESeq2 and edgeR analysis in response to IHNV exposure or kidney infection was 25,294 (42 % prefiltered dataset) and 19,636 (32 % prefiltered dataset), respectively, (Fig. 4b). For the purpose of this manuscript, we give only brief focus to these differential comparisons, which encompass Group 3 (mock) versus 4 (IHNV challenged but without qPCR detectable kidney infections) and Group 3 verses 5 (IHNV challenged with detectable kidney infections). A more complete analysis of the IHNV responsiveness of this transcriptome is to follow in a separate manuscript.

Prior infection with PRV appeared to have a negligible effect on the transcriptional response of head kidney following exposure or infection with IHNV. Only two DEGs specific to PRV/IHNV coinfection were identified by combined edgeR and DESeq2 analysis (Fig. 4c; Table 2).

### qPCR confirmed elevated transcription of Ependymin and not classic antiviral or inflammatory genes during early PRV infection

**Fig. 5:**
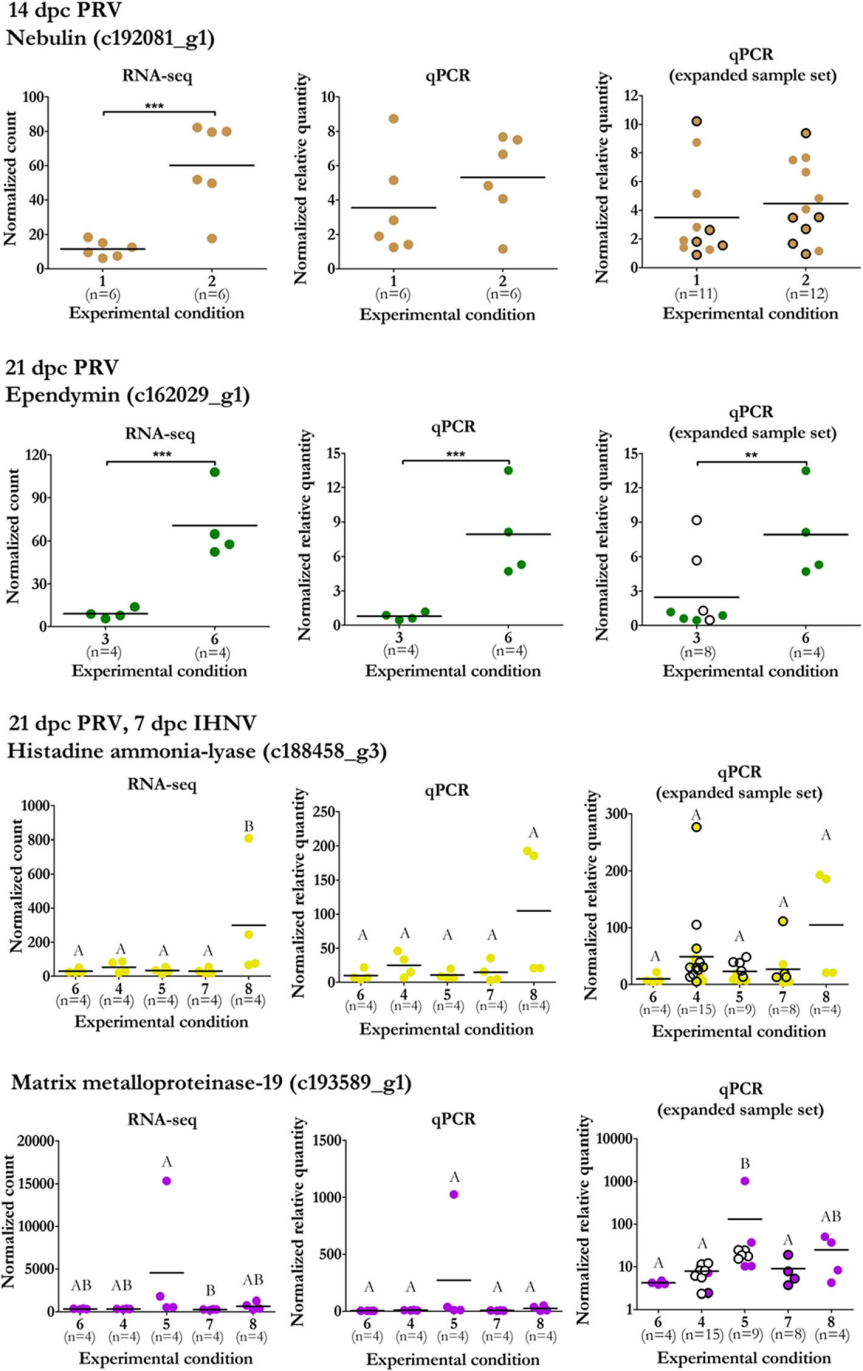
Comparative RNA-seq and qPCR calculated expression of PRV responsive genes. For each select PRV responsive unigene, DESeq2 normalized counts (*left*) are presented alongside relative quantities assessed by qPCR from the same samples used to prepare RNA-seq libraries (*middle*) or with additional samples not incorporated into RNA-seq analysis where available (*right*) normalized to two stable reference genes. Samples not included in RNA-seq but with identical condition-specific parameters are indicated (*black border*). For groups which received mock PRV injections, samples which received L-15 media injections (*open circles*) are distinguished from those which received blood homogenate injections (*filled circles*). Significant differential expression between experimental groups are indicated at *p* < 0.05 (*), 0.01 (**) and 0.001 (***) by t-test or at *p* < 0.05 by ANOVA with post hoc Tukey test where appropriate. Group 1: mock; Group 2: PRV; Group 3: mock; Group 4: IHNV exposed qPCR-; Group 5: IHNV exposed qPCR+; Group 6: PRV; Group 7: PRV/IHNV exposed qPCR-; Group 8: PRV/IHNV exposed qPCR+ (Table 1)

Of the 19 RNA-seq unigenes identified with putative PRV responsiveness (Table 2), we selected four of the six unigenes with protein-coding annotation for qPCR validation: Nebulin (*Nebu*) at 14 dpc PRV, Ependymin (*Epd*) at 21 dpc PRV, and Matrix Metalloproteinase-19 (*MMP-19*) and Histidine Ammonia-lyase (*HAL*) at 21 dpc PRV/7 dpc IHNV. Expression of these genes by qPCR in reference to two stable housekeeping genes (*βactin* and *EF1α*) showed that *Epd* had significant differential regulation in response to PRV at 21 dpc. This validation was further confirmed in an expanded sample set which included an additional four L-15 mock infected controls (Fig. 5). Differential regulation of the other three selected genes was not supported by qPCR in either the original or an expanded sample set.

**Fig. 6:**
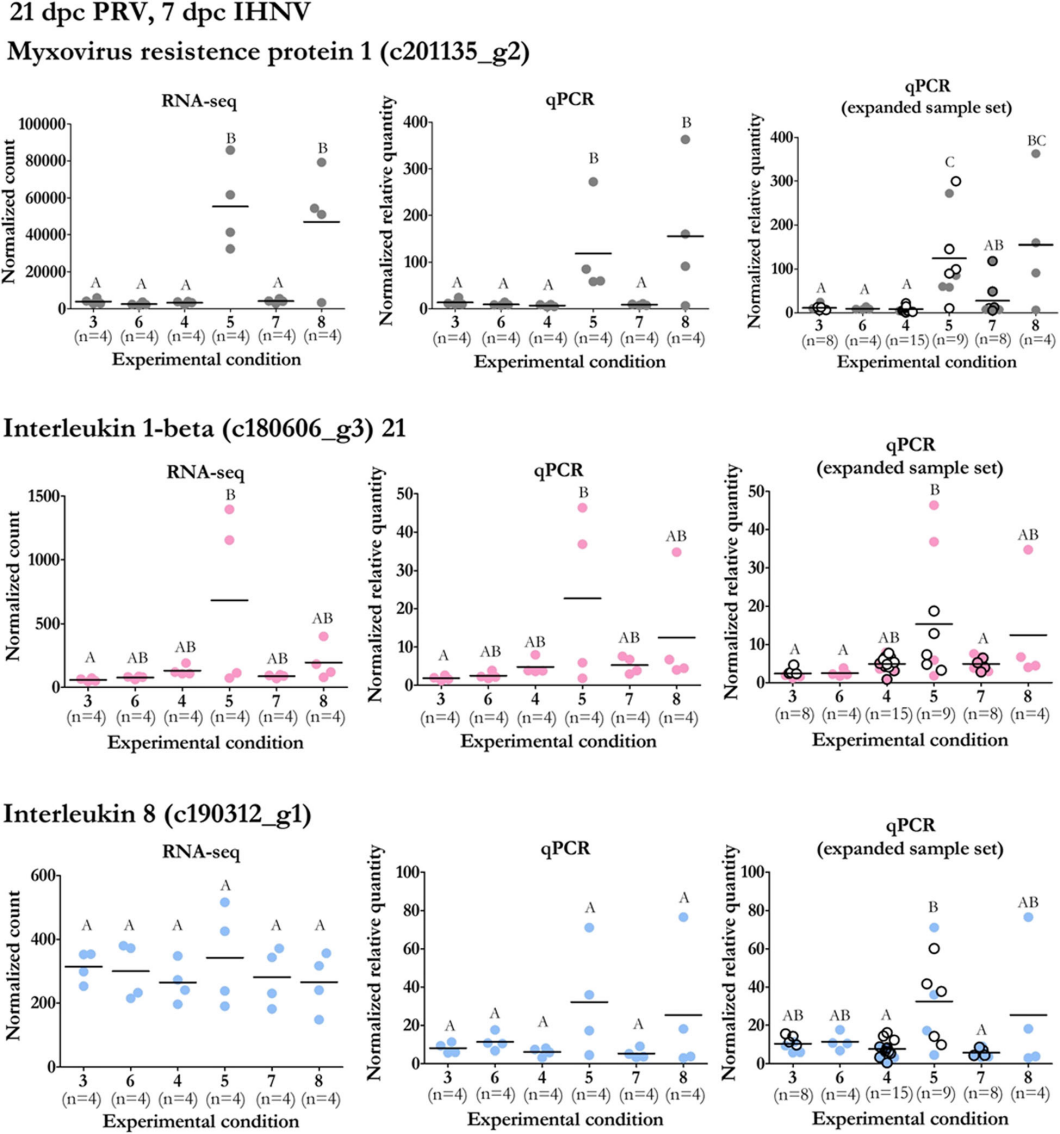
RNA-seq and qPCR expression of select antiviral and inflammatory response genes. DESeq2 normalized counts of three immune response genes (*left*) are presented alongside relative quantities for the same samples assessed by qPCR (*middle*) or with additional samples where available (*right*). Samples with identical condition-specific parameters to those tested by RNA-seq are indicated (black border). For groups which received mock PRV injections, samples which received L-15 media injections (*open circles*) are distinguished from those which received blood homogenate injections (*filled circles*). Significant differential expression between groups is indicated at *p* < 0.5 by ANOVA and post hoc Tukey test. Group 3: mock; Group 4: IHNV exposed qPCR-; Group 5: IHNV exposed qPCR+; Group 6: PRV; Group 7: PRV/IHNV exposed qPCR-; Group 8: PRV/IHNV exposed qPCR+ (Table 1)

In addition, we further confirmed the apparent lack of antiviral or inflammatory responses to infection with PRV by examining the gene expression of three immune-associated proteins: the classical interferon viral response protein Myxovirus resistence protein 1 (*Mx*), the inflammatory cytokine Interleukin 1β (*IL-1β*), and the inflammatory chemokine Interleukin 8 (*IL-8*) at 21 dpc PRV. There was no significant difference in the expression of these genes in response to PRV infection alone (Groups 3 and 6) or when PRV infected fish were exposed but not infected with IHNV (Groups 4 and 7) (Fig. 6). There was a high level of variability in expression of all three genes in fish that were infected with IHNV regardless of their PRV infection status. When compared to Groups 3, 4, 6 and 7, there was significantly higher expression of *Mx* in IHNV infected Sockeye (Groups 5 and 8) regardless of PRV infection status, whereas the expression of *IL-1β* and *IL-8* was not significantly different as a result of IHNV exposure and appeared more responsive to those kidney samples with detectable IHNV infections.

During a preliminary DEG analysis, we used DESeq (older version) rather than DESeq2 in conjunction with edgeR to screen unigenes without low-count filtration. This resulted in an alternate set of putative PRV responsive DEGs than those subsequently identified by DESeq2 and edgeR using the prefiltered unigene dataset presented above. Specifically, this preliminary analysis identified 14 and 67 DEGs at 14 and 21 dpc PRV, respectively (Additional file 8). No DEGs were shared between 14 and 21 day time points in this preliminary analysis, and only two unigenes were common to both the preliminary and final analyses: *Nebu* and *Epd*, for which only *Epd* was validated by qPCR (Fig. 5). Nevertheless, nine additional unigenes of potential immune relevance were also screened by qPCR in an expanded early time point sample set (2–14 dpc PRV). Of these, three genes were identified with putative PRV responsiveness for at least one time point, although all were below a twofold variation from mock injected controls and were not pursued further in this study (Additional file 8). A fourth gene, cluster of differentiation-2 (*CD2*), also showed potential PRV responsiveness at 21 dpc as it was not observed to be expressed in any of the four PRV challenged kidney samples but was present in three of the four mock challenged samples. However, further exploration of *CD2* responsiveness to PRV was not undertaken in this study due to its inconsistent expression in kidney samples across all temporal treatment groups (Additional file 8).

### Ependymin expression was confined to early PRV infection in kidney but not blood and was followed by a later antiviral response

**Fig. 7:**
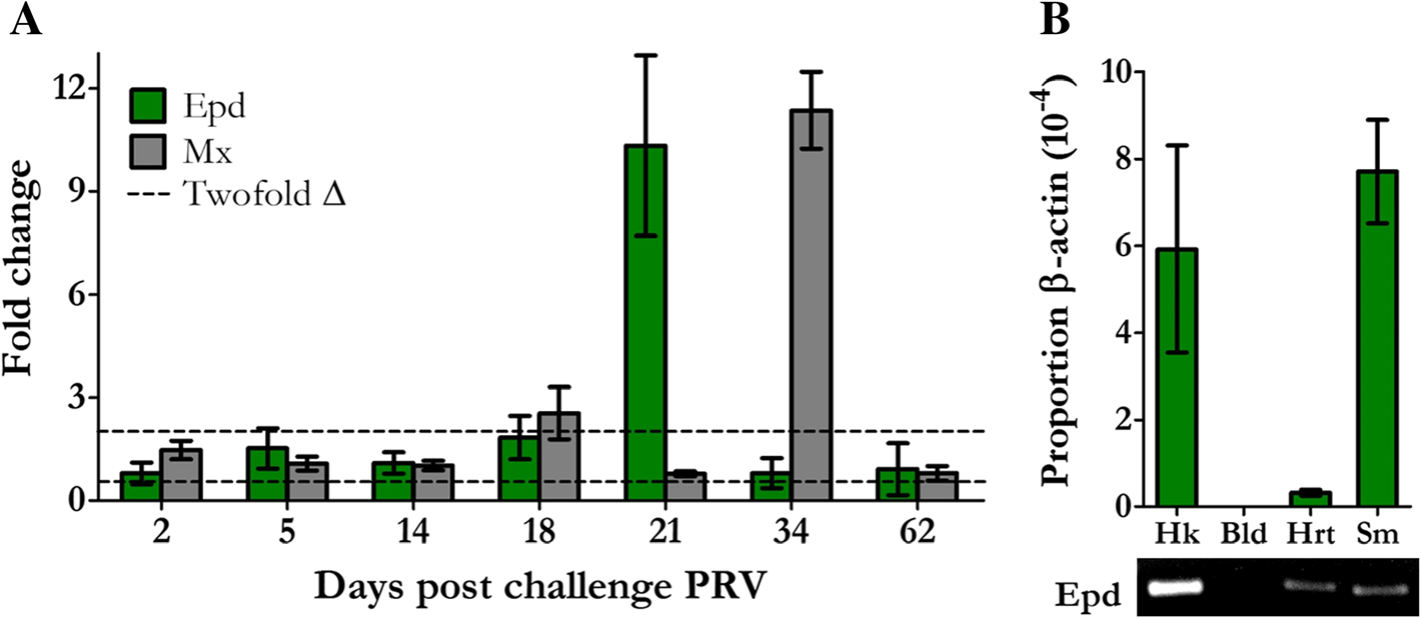
Expression of Ependymin and Myxovirus resistance protein following PRV challenge. **a** Mean (± SE) fold change in transcriptional expression of Ependymin (*Epd*) and Myxovirus resistance protein (*Mx*) in PRV infected samples is presented relative to time-matched mock infected controls at seven discrete time points following PRV injection challenge. The dotted lines represent a minimum fold change (Twofold) suggestive of biological significance. **b** Constitutive expression of *Epd* in proportion to *β-actin* is provided for head kidney (Hk), blood (Bld), heart (Hrt), and skeletal muscle (Sm) from PRV and IHNV free Sockeye salmon (*n* = 6). Visualization of qPCR products for *Epd* following 35 amplification cycles from comparable amounts of total extracted RNA (~1.5 μg) is also provided

Quantitative PCR confirmed elevated *Epd* transcription but not *Mx* at 21 dpc PRV. Nevertheless, we further examined the transcription of these genes at both earlier and later time points in an attempt to better understand the temporal significance of their transcription (Fig. 7a). This identified that increased expression of *Epd* was only observed at the 21 dpc sampling time point. The expression of *Mx* during PRV infection remained comparable to mock infected controls for sampling time points out to 21 dpc; a period marked by significant increases in systemic viral transcripts representing the major period of dissemination which we designate as the early phase of infection. However, at 34 dpc PRV an approximate eleven-fold increase in *Mx* was observed in PRV infected individuals (Fig. 7a). This time point corresponds to the highest levels of PRV L1 transcripts noted in this study which we believe represents a mature phase of viral infection, although it should be noted that a period of 4 weeks elapsed between this and the subsequent sampling event which obscures precise resolution for the timing of the peak in viral loads. Nevertheless, the elevated expression of both these genes was observed to be transient during persistent PRV infection as neither *Epd* nor *Mx* was differentially expressed at the termination of the 62 day PRV challenge.

To identify if PRV associated *Epd* expression was blood or kidney specific, we further attempted to evaluate *Epd* expression by qPCR in peripheral blood of sampled fish used to demonstrate kidney expression. No amplification occurred in 35–40 cycles of qPRC in either PRV infected or non-infected blood cell populations (data not shown). Additional qPCR analysis of tissues from six non-PRV infected individuals revealed that *Epd* was constitutively expressed in the kidney, heart and skeletal muscle of Sockeye salmon but not in peripheral blood (Fig. 7b).

## Discussion

Sockeye salmon have previously been shown to develop considerable blood and kidney infections following cohabitation with PRV infected Atlantic salmon [17]. Here we confirm that Sockeye develop similar infections following i.p. injection of PRV prepared from infected Atlantic salmon blood. As with previous work, no weight loss, morbidity or pathology could be attributed to the presence of PRV in Sockeye during this study. These results supports a growing body of evidence that PRV is infectious but non-pathogenic to Pacific salmon in western North America [13, 14, 17]. Nevertheless, even in Norway where PRV has been associated with disease in Atlantic salmon, the widespread prevalence of virus in non-diseased fish, sometimes at high viral load [5, 12] indicates that much is still unknown with regard to PRV pathogenicity; particularly concerning mechanisms involved for inducing pathology.

In this study, we used RNA-seq technologies to develop a reference transcriptome for Sockeye salmon head kidney. The Trinity *de novo* assembly platform used here has been shown to resolve closely paralogous genes and polymorphic transcripts in a broad range of organisms [24], including an Atlantic salmon leukocyte cell line [42], and is currently considered the forerunning tool for vertebrate transcriptome assembly in the absence of a reference genome [43]. To our knowledge this is the first RNA-seq transcriptome dataset for Sockeye salmon, and provides an important genetic reference for this ecologically and economically important Pacific salmon species. The total number of unigenes identified here (326,410) is comparable to the number of unigenes reported from the *de novo* assembly of a multi-tissue transcriptome of catfish (370,798) [44]; and although a higher proportion of catfish genes was initially identified as protein coding relative to Sockeye (25 % vs 15 %), this is likely due to differences in categorizing isoforms during assembly [24]. Indeed, the number of putative protein coding genes identified herein (47,429) is consistent with those identified in the notochord transcriptome (55,775) [45] and whole-body transcriptomes (41,735-43,654) [46] of Atlantic salmon using genome guided assembly. Additionally, an all-by-all blastn search of the current Sockeye sequences against the latest salmon repeat database provided by the University of Victoria consortium for Genomics Research on all Salmon Project (cGRASP; http://web.uvic.ca/grasp/) revealed at least 33,456 non-redundant unigenes (Additional file 9). This is higher than the quantity of unique protein encoding genes identified within the catfish transcriptome (25,144) [44] and is consistent with the 4R salmonid-specific whole-genome duplication [47, 48] (Additional file 9).

We further identify the Sockeye kidney transcriptome to be highly responsive to the aquatic single-stranded RNA rhabdovirus IHNV. A large degree of variance in global gene expression could be attributed to IHNV infection at 7 dpc and more than 20,000 unigenes showed putative differential expression following exposure to waterborne virus. This included significant induction of antiviral (*Mx*) and inflammatory (*IL-1β*) associated genes as assessed by qPCR, which is consistent with previous observations of IHNV infections of rainbow trout (*Oncorhynchus mykiss*) [49], and is suggestive that both type-I interferon and NF-κB cellular response pathways are initiated during early IHNV kidney infection. Nevertheless, most of the global changes in Sockeye gene expression caused by IHNV have yet to be explored and we are currently expanding our investigations regarding the IHNV responsiveness of this transcriptome. We intend to provide further details regarding cell signalling pathways and examine the importance of differentially regulated non-protein encoding transcripts in a subsequent manuscript.

In stark contrast to the well-developed responses generated against IHNV, PRV appeared to cause almost no kidney-specific gene expression changes in Sockeye at either 2 or 3 weeks post challenge (wpc). The 17 putative PRV-associated DEGs identified in this study account for only 0.03 % of the unigenes used in the analysis and none of the genes identified as differentially expressed at 14 dpc were shared with 21 dpc. The majority of DEGs identified appear to be non-protein encoding and for those which had blastx annotations (Laminin subunit beta-1, Nebulin, Ependymin and 18 s rRNA) only Ependymin has currently been validated by qPCR.

Ependymin is a glycoprotein that was originally discovered in the cerebrospinal fluid of goldfish [50] and is believed to be involved in learning processes [51] and behaviour [52]. More recently however, a number of *Epd* paralogues have been identified in a variety of other vertebrate organs, particularly those of fish, although their functions are largely unknown [53]. Here, we identify that at least one *Epd* gene is constitutively expressed in kidney, heart, and skeletal muscle of Sockeye salmon but not in peripheral blood (Fig. 7). We also identified this gene to be the only qPCR-validated differentially expressed protein coding gene responsive to early PRV infection in head kidney tissues. Interestingly, *Epd* expression has also been shown to be upregulated in the liver of Atlantic salmon following exposure to the bacterial pathogen *Aeromonas salmonicida* [54], in the liver of medaka (*Oryzias latipes*) exposed to dioxin [55], and in the distal intestine of Atlantic salmon fed camelina-derived feeds [56]; suggesting the transcriptional regulation of *Epn* may be involved in immunological, inflammatory, or cellular repair responses. However, as neither immunological, inflammatory, cellular repair, nor indeed any other transcriptional pathway appeared to correspond to *Epn* expression in this study its biological role in response to PRV appears limited.

In a previous PRV challenge study with Atlantic salmon, we identified only modest and/or questionable increases (less than fourfold) in gene expression of the classic antiviral response protein *Mx* during early PRV infection following i.p. injection challenge in both blood and kidney during log-linear viral amplification [17]. Here we confirm a lack of an *Mx* transcriptional response during early PRV infection in Sockeye kidney and further identify a lack of other antiviral transcriptional responses during this period of significant increasing viral load. Together, these studies indicate that PRV is able to evade or block host responses during primary infection, amplification and hematogenous dissemination within salmonids of western North America during non-pathogenic infection.

Interestingly, an absence of host antiviral signalling during early systemic PRV infections may not be exclusive to non-pathogenic conditions. Dahle *et al.* [9] examined the transcriptional response of Atlantic salmon erythrocytes to infection with PRV using samples obtained from a cohabitation challenge conducted by Finstad *et al.* [7] which resulted in a first detection of PRV at 4 wpc and histological signs of HSMI at 7 wpc when blood PRV loads were at their maximum. Microarray analysis conducted on peripheral erythrocyte samples collected at 5 and 7 wpc reported upregulation of a large number of viral responsive genes and down-regulation of non-immune related genes in PRV infected groups at both time points; however, qPCR validation of antiviral genes associated with the interferon response (*IFN-1α*, *IRF-1*, *RIG-1*, *Mx* and *PKR*) as well as the immuno-regulatory genes Interleukin-10 and Suppressor of Cytokine Signaling-1 did not identify significant responsiveness of these genes at 5 wpc relative to controls. At 7 wpc, when 4/6 fish sampled had histopathological signs of HSMI and PRV loads were highest, significant induction of these genes (except PKR) was observed although variability between individuals appeared high [9].

In our present study, we identified increased *Mx* expression in kidney tissues only once peak viral loads were reached, which in conjunction with the work of Dahle *et al.* [9], suggests that an antiviral response against PRV is initiated late during the mature phase of PRV infection and can occur in both the presence and absence of inflammatory associated disease. However, without disease manifestations, we observed that *Mx* transcription was not maintained and returned to a comparable control level by 9 weeks post challenge (Fig. 7a). In another study, Johansen *et al.* [10] examined the transcriptional response of Atlantic salmon cardiac tissues during a mature phase of PRV infection over a period of 5 to 11 wpc which used an injection challenge of PRV inoculum made from hearts of Atlantic salmon experiencing HSMI. This work appeared to encompass a period after peak PRV infections were reached, as decreasing quantities of PRV transcripts were identified in heart tissues from the first sampling at 5 wpc through to the end of the experiment at 11 wpc when only one out of five fish tested positive for PRV. Histopathological signs of HSMI were first evident at 6 wpc (1/6 fish), became prevalent at 7 wpc (6/6 fish), then declined at the end of the 11 week trial (2/6 fish). However, even though PRV loads were in decline and many heart tissues appeared to be in the process of clearing PRV infection, expression of viral responsive DEGs was reported to be consistently elevated in challenged heart tissues relative to time-0 controls throughout this period. Although different tissues make direct comparisons difficult, these results provide counterpoint to the brief antiviral responsiveness of Sockeye kidney to PRV in the absence of disease, and suggest that HSMI may perpetuate antiviral signalling as a direct result of pathology in infected tissues.

Currently, the only study that has observed significant host transcriptional responsiveness to PRV during early PRV amplification was recently reported by Johansen *et al*. [57] who challenged Atlantic salmon parr and smolts with PRV through cohabitation in freshwater and seawater, respectively, using PRV source material from an outbreak of HSMI. The challenge resulted in heart lesions typical of HSMI in both life history stages at 8 weeks post cohabitation and many antiviral responsive genes were shown to be unregulated in kidney and spleen by microarray analysis during early PRV infection (particularly in parr) for which some genes were confirmed to be significantly elevated by qPCR. However, both microarray and qPCR analysis identified significant induction of antiviral genes at 4 wpc even though PRV transcripts were not detected by qPCR until 6 wpc, which makes the relationship of their expression in reference to PRV very difficult to interpret.

Why Sockeye salmon infected with PRV have such a limited transcriptional response in the presence of increasing viral loads is unclear; however, many reoviruses are known to use blood as a means for disseminating from a port of entry to target tissues and leukocytes, erythrocytes and platelets have all been shown to be susceptible to at least one form of reoviral infection in a wide range of host organisms (reviewed by [58]). Nevertheless, the presence of reovirus in blood is not usually associated with anemia or leukopenia, and to our knowledge reoviruses have not been shown to induce antiviral responses *in vivo* directly from blood cells due to infection. In our current study we did not observe anemia due to the presence of PRV (Additional file 4) which has also been noted previously in other non-pathogenic PRV infections of Atlantic and Pacific salmon [13, 17] as well as during infections associated with HSMI [7, 11]. In contrast, dissemination of many pathogenic orthoreoviruses into target tissues quickly leads to host immune responses and pathology. For example, induction of host immune responses leading to cellular apoptosis is considered the major cause for mammalian reovirus (MRV) encephalitis and myocarditis, which appears to be initiated shortly after viral cell entry and before the core particles become transcriptionally active in these target cells [59, 60]. Thus, the general lack of cellular responses and apoptosis signalling in blood compared to tissues following mammalian reoviral infection is suggestive of a fundamental difference in the way blood cells and target tissue cells interact with this virus. We hypothesize that PRV also interacts differently with blood cells compared to tissue cells, and that PRV can evade antiviral responses during erythrocyte entry and replication *in vivo*. Following primary replication in erythrocytes, we suspect the dissemination of virus into resident tissue cells causes host antiviral responses for which *Epd* may be involved. Still, erythrocytes have been shown to mount antiviral responses against PRV *ex vivo* [11] and further work will be required to identify a mechanism to explain the variability for PRV to induce host cell responses during natural infection.

Differences in cell and/or tissue type interaction with PRV also does not explain the presence or absence of HSMI following PRV infection, as PRV loads have been observed to reach comparable levels with or without pathology in both heart and skeletal muscle [6, 17]. One potential explanation for this disparity may involve genetic variation between PRV isolates. Siah *et al.* [3] recently conducted a phylogenetic analysis of all segments of the PRV obtained from western North America as well as selected Norwegian and Chilean PRV genomes. With the exception of segment S4, samples of North American PRV clustered separately from the Norwegian and Chilean samples indicating that there are genetic differences across the genome between Pacific, Norwegian and Chilean forms of PRV. In the case of mammalian reoviruses, small changes in the genome can be reflected in dramatic changes in infectivity and virulence [61, 62]. For example, Doyle *et al.* [61] identified that a single amino change in the ơ3 protein, which affects outer capsid stability, was sufficient to increase replication, host to host spread, and virulence in mice. Based on the analysis of samples of PRV from Norway, Wessel et al. [11] determined that the ơ3 protein had a dsRNA binding function similar to that reported for the ơ3 protein of mammalian orthoreovirus. The dsRNA-binding activity of the MRV ơ3 protein inhibits the induction of type-1 IFN and activation of protein kinase R (*PKR*) which through its interaction with translation initiation factor (*eIF2α*) inhibits cellular mRNA translation, thereby, limiting viral protein production. Siah *et al.* [3] compared the predicted structure of the North American PRV ơ3 protein to predicted ơ3 proteins based on Norwegian/Chilean sequences. These authors identified an amino acid difference which results in a predicted change in secondary structure from an “alpha-helix” in Norwegian/Chilean PRV source to a “sheet structure” in North American PRV sequences. These authors also identified an amino acid substitution in the μ1 protein between North American and Norwegian/Chilean PRV which results in a predicted change in protein structure. Whether these predicted changes in secondary structure results in changes of function for these proteins and or virulence differences between these PRV sources is unknown.

Epigenetic host factors may also provide a possible explanation for regional or stock inconsistency regarding PRV associated disease. In this study, the majority of unigenes that were expressed in response to PRV infection were non-coding. Additional investigation and validation for the putative PRV responsive long non-coding RNAs (lncRNAs) identified in this study may therefore prove valuable, as lncRNAs have been shown to play critical roles in epigenetic transcription silencing [63, 64] and can have post-translational abilities to alter protein function in mammals [65]. A number of lncRNAs have also been speculated to be important during infectious salmon anemia virus (ISAV) infection of Atlantic salmon [66], although the functions for either PRV-responsive host lncRNAs identified in this study or ISAV-responsive lncRNAs identified previously are so far unknown.

Another potential factor for altering virulence during PRV infection could be the involvement of a coinfecting pathogen. A number of significant human diseases are known to be caused or exacerbated as a result of coinfections involving viral pathogens [67–71], and infection by avian reovirus in chickens has been shown to substantially increase anemia caused by chicken anemia virus during dual infection [72]. Alternatively, a coinfecting agent may provide protection against other viral infection; such as in the increased survival afforded to chum salmon reovirus (CSV) or infectious pancreatic necrosis virus (IPNV) infected rainbow trout during lethal IHNV superinfection [73, 74]. In this study, PRV did not protect Sockeye against superinfection IHNV. This is not entirely surprising, as the induction of interferon antiviral responses is usually the mechanism for protection during viral coinfections and is likely responsible for the previous reports of IHNV protection in rainbow trout afforded by CSV or IPNV mentioned above [75]. As PRV had little to no effect on systemic or kidney specific cellular signalling for the first 3 weeks following exposure which included the first week (peak load) of IHNV superinfection, a similar mechanism for protection seems unlikely to be generated from PRV during this period. Nevertheless, we hypothesize timing may be critical with regard to this type of putative protection, and suspect that exposure to IHNV following peak PRV dissemination which causes host antiviral activation (in this study, a brief period starting at 4 wpc) may indeed contribute to a reduced virulence of IHNV if exposure occurred during this time. Further work in identifying such protection may warrant additional investigation; however any putative protection would likely be short lived, as antiviral responses generated against PRV in this study abated by 9 wpc in the absence of PRV associated disease.

Conversely, one of the more surprising observations of this study was the lack of protection afforded by host antiviral responses against IHNV to inhibit PRV transcription and dissemination. PRV transcriptional loads continued to increase at comparable rates in both blood and kidney with or without the considerable transcriptomic changes induced by IHNV. In a previous Norwegian study, chronic IPNV infections also did not significantly alter PRV transcription, nor was vaccination with bacterial and IPNV antigen 8 weeks before PRV exposure able to reduce PRV loads at 7 wpc [76]. Collectively, these data suggest that although PRV does not appear to induce a significant antiviral state during early dissemination, the activation of host antiviral responses during this time appears inconsequential to PRV transcription in either the blood or kidney.

## Conclusions

Here we demonstrate that PRV causes minimal changes to the Sockeye salmon head kidney transcriptome during the first 3 weeks of infection following injection. Viral loads increased substantially during this period in both blood and kidney tissues which indicate that PRV has the ability to evade or block host recognition during hematogenous dissemination and early replication within the Sockeye salmon. In this instance, evasion rather than active blocking of host responses is more likely, as PRV had no measured impact on the substantial host transcriptional responses generated against superinfection with IHNV during this period.

Robust host transcriptome responses that included activation of classic antiviral pathways generated during IHNV superinfection did not affect increases in PRV transcriptional load. The mechanisms for why antiviral responses fail to impact PRV transcription, induce erythrocyte/renal cell apoptosis, or lead to pathology in Sockeye salmon are still unclear; however we suspect variations in the way PRV interacts with different cell types may be important. Here we identified a late increase in *Mx* expression at 4 wpc during a period of peak viral loads in kidney tissue which was preceded by an increased expression of *Epd* at 3 wpc in PRV infected kidneys but not blood. Although the role of *Epd* in PRV infection is currently unknown, its previous association with stress/immune functions in other fish species and its lone transcriptional regulation during early PRV infection in Sockeye suggests an important putative role in host PRV responsiveness and warrants further investigation.

The reason for the very limited transcriptional response in Sockeye salmon to PRV within the first 3–4 weeks of infection remains unclear. Although we hypothesize that disparities in the way PRV interacts with different cell types may explain some aspects regarding host responsiveness to PRV, there are many factors involved in PRV pathogenesis which make comparisons of current studies difficult. For example, some transcriptional differences observed in response to PRV can likely be attributed to differences in host species (Sockeye vs. Atlantic salmon). This study also focused on the head kidney which may have a different transcriptional profile when compared to heart tissues. It is also possible that genotypic differences exist between North American PRV and the types of PRV that are associated with strong transcriptional responses and the presentation of the HSMI disease state in Norway. Lastly, the experiment that we are reporting was conducted in a controlled environment with optimal water conditions. Under these conditions, it appears that PRV is of low virulence to Sockeye salmon and that carriers of PRV are not at a disadvantage when it comes to superinfection with IHNV. Whether these results will hold under less optimal more stressful conditions such as those experienced in the field warrant further consideration.

## Additional files

Additional file 1: Oligonucleotide primers and probes used in real time qPCR analyses. (PDF 153 kb)
Additional file 2: Command and session information used to generate edgeR and DESeq2 differential gene expression analysis. (RAR 8377 kb)
Additional file 3: Histopathological findings in Sockeye salmon exposed to Piscine orthoreovirus by injection and/or IHNV by cohabitation challenge. (PDF 262 kb)
Additional file 4: Sample inventory detailing fish identification number, treatment group, sampling date, and associated metrics for each fish collected from challenge experiments are provided along with PRV and IHNV challenge morbidity log. (XLSX 68 kb)
Additional file 5: All contiguous sequences (putative transcripts) generated from *de novo* assembly of Sockeye salmon head kidney tissues. (RAR 108706 kb)
Additional file 6: Associated UniProt 2013_11 protein database annotation of all Sockey salmon head kidney unigene components. (RAR 14953 kb)
Additional file 7: RSEM derived gene counts for all generated component unigenes of Sockeye salmon head kidneys identified by RNA-seq. (RAR 8337 kb)
Additional file 8: Putative DEGs identified by preliminary DESeq and edgeR analysis and subsequent qPCR validation. (PDF 1454 kb)
Additional file 9: Non-redundant putative transcripts identified using the University of Victoria consortium of genomics research on all salmon repeat database. (RAR 16480 kb)

## Abbreviations

ARP: Acidic ribosomal protein
CD2: Cluster of differentiation 2
CSV: Chum salmon reovirus
DEG: Differentially expressed gene
Dyn: Dynein
EF1-α: Elongation factor-1 alpha
EPC: Epithelioma papulosum cyprini cell line
Epd: Ependymin
FDR: False discovery rate
HAL: Histadine ammonia-lyase
HBSS: Hank’s balanced salt solution
HSMI: Heart and skeletal muscle inflammation
IHN: Infectious hematopoietic necrosis
IHNV: Infectious hematopoietic necrosis virus
IL-1β: Interleukin 1 beta
IL-8: Interleukin 8
IPNV: Infectious pancreatic necrosis virus
ISAV: Infectious salmon anemia virus
lncRNA: Long-strand non-coding RNA
MDA: Multidimensional scaling analysis
MMP-19: Matrix metalloproteinase-19
MRV: Mammalian reovirus
MS222: Tricaine methanesulfonate
Mx: Myxovirus resistence protein
Nebu: Nebulin
PCA: Principal component analysis
PRV: Piscine reovirus
qPCR: Real-time quantitative polymerase chain reaction
RNA-seq: Next-generation RNA sequencing
RSEM: RNA-seq by Expectation-Maximization
β-Actin: beta-actin

## References

[1] Palacios G, Lovoll M, Tengs T, Hornig M, Hutchison S, Hui J, Kongtorp R-T, Savji N, Bussetti AV, Solovyov A (2010). Heart and skeletal muscle inflammation of farmed salmon is associated with infection with a novel reovirus. PLoS One.

[2] Markussen T, Dahle MK, Tengs T, Løvoll M, Finstad ØW, Wiik-Nielsen CR, Grove S, Lauksund S, Robertsen B, Rimstad E (2013). Sequence analysis of the genome of piscine orthoreovirus (PRV) associated with heart and skeletal muscle inflammation (HSMI) in Atlantic salmon (Salmo salar). PLoS One.

[3] Siah A, Morrison DB, Fringuelli E, Savage P, Richmond Z, Johns R, Purcell MK, Johnson SC, Saksida SM (2015). Piscine reovirus: genomic and molecular phylogenetic analysis from farmed and wild salmonids collected on the Canada/US Pacific Coast. PLoS One.

[4] Lovoll M, Wiik-Nielsen J, Grove S, Wiik-Nielsen CR, Kristoffersen AB, Faller R, Poppe T, Jung J, Pedamallu CS, Nederbragt AJ (2010). A novel totivirus and piscine reovirus (PRV) in Atlantic salmon (Salmo salar) with cardiomyopathy syndrome (CMS). Virol J.

[5] Lovoll M, Alarcon M, Bang Jensen B, Taksdal T, Kristoffersen AB, Tengs T (2012). Quantification of piscine reovirus (PRV) at different stages of Atlantic salmon Salmo salar production. Dis Aquat Organ.

[6] Finstad OW, Falk K, Lovoll M, Evensen O, Rimstad E (2012). Immunohistochemical detection of piscine reovirus (PRV) in hearts of Atlantic salmon coincide with the course of heart and skeletal muscle inflammation (HSMI). Vet Res.

[7] Finstad OW, Dahle MK, Lindholm TH, Nyman IB, Lovoll M, Wallace C, Olsen CM, Storset AK, Rimstad E (2014). Piscine orthoreovirus (PRV) infects Atlantic salmon erythrocytes. Vet Res.

[8] Mikalsen AB, Haugland O, Rode M, Solbakk IT, Evensen O (2012). Atlantic salmon reovirus infection causes a CD8 T cell myocarditis in Atlantic salmon (Salmo salar L.). PLoS One.

[9] Dahle MK, Wessel Ø, Timmerhaus G, Nyman IB, Jørgensen SM, Rimstad E, Krasnov A (2015). Transcriptome analyses of Atlantic salmon (Salmo salar L.) erythrocytes infected with Piscine orthoreovirus (PRV). Fish Shellfish Immunol..

[10] Johansen L-H, Thim HL, Jørgensen SM, Afanasyev S, Strandskog G, Taksdal T, Fremmerlid K, McLoughlin M, Jørgensen JB, Krasnov A (2015). Comparison of transcriptomic responses to pancreas disease (PD) and heart and skeletal muscle inflammation (HSMI) in heart of Atlantic salmon (Salmo salar L). Fish Shellfish Immunol..

[11] Wessel Ø, Olsen CM, Rimstad E, Dahle MK (2015). Piscine orthoreovirus (PRV) replicates in Atlantic salmon (Salmo salar L.) erythrocytes ex vivo. Vet Res.

[12] Garseth AH, Fritsvold C, Opheim M, Skjerve E, Biering E (2013). Piscine reovirus (PRV) in wild Atlantic salmon, Salmo salar L., and sea-trout, Salmo trutta L., in Norway. J Fish Dis.

[13] Garver KA, Marty GD, Cockburn SN, Richard J, Hawley LM, Müller A, Thompson RL, Purcell MK, Saksida S (2016). Piscine reovirus, but not Jaundice Syndrome, was transmissible to Chinook Salmon, Oncorhynchus tshawytscha (Walbaum), Sockeye Salmon, Oncorhynchus nerka (Walbaum), and Atlantic Salmon, Salmo salar L. J Fish Dis.

[14] Marty GD, Morrison DB, Bidulka J, Joseph T, Siah A (2015). Piscine reovirus in wild and farmed salmonids in British Columbia, Canada: 1974–2013. J Fish Dis.

[15] Kibenge MJ, Iwamoto T, Wang Y, Morton A, Godoy MG, Kibenge FS (2013). Whole-genome analysis of piscine reovirus (PRV) shows PRV represents a new genus in family Reoviridae and its genome segment S1 sequences group it into two separate sub-genotypes. Virol J.

[16] Department of Fisheries and Oceans Canada. Assessment of the Occuurrence, Distribution and Potential Impacts of Piscine Reovirus on the West Coast of North America. http://publications.gc.ca/site/eng/9.809662/publication.html; Fisheries and Oceans Canada, 2015.

[17] Garver KA, Johnson SC, Polinski MP, Bradshaw JC, Marty GD, Snyman HN, Morrison DB, Richard J (2016). Piscine orthoreovirus from western North America is transmissible to Atlantic salmon and Sockeye salmon but fails to cause Heart and Skeletal Muscle Inflammation. PLoS One.

[18] Yamamoto T, Clermont T (1990). Multiplication of infectious hematopoietic necrosis virus in rainbow trout following immersion infection: organ assay and electron microscopy. J Aquat Anim Health.

[19] Garver KA, Mahony AA, Stucchi D, Richard J, Van Woensel C, Foreman M (2013). Estimation of parameters influencing waterborne transmission of infectious Hematopoietic Necrosis Virus (IHNV) in Atlantic Salmon (Salmo salar). PLoS One.

[20] Steven A (1990). The haematoxyline. The theory and practice of histological techniques.

[21] Purcell MK, Thompson RL, Garver KA, Hawley LM, Batts WN, Sprague L, Sampson C, Winton JR (2013). Universal reverse-transcriptase real-time PCR for infectious hematopoietic necrosis virus (IHNV). Dis Aquat Organ.

[22] Aranda PS, LaJoie DM, Jorcyk CL (2012). Bleach gel: a simple agarose gel for analyzing RNA quality. Electrophoresis.

[23] Haas BJ, Papanicolaou A, Yassour M, Grabherr M, Blood PD, Bowden J, Couger MB, Eccles D, Li B, Lieber M (2013). De novo transcript sequence reconstruction from RNA-seq using the Trinity platform for reference generation and analysis. Nat Protoc.

[24] Grabherr MG, Haas BJ, Yassour M, Levin JZ, Thompson DA, Amit I, Adiconis X, Fan L, Raychowdhury R, Zeng Q (2011). Full-length transcriptome assembly from RNA-Seq data without a reference genome. Nat Biotechnol.

[25] Bolger AM, Lohse M, Usadel B. Trimmomatic: a flexible trimmer for Illumina Sequence Data Bioinformatics. 2014. doi:10.1093/bioinformatics/btu170. 10.1093/bioinformatics/btu170 PMC4103590 24695404

[26] Ye J, Fang L, Zheng H, Zhang Y, Chen J, Zhang Z, Wang J, Li S, Li R, Bolund L (2006). WEGO: a web tool for plotting GO annotations. Nucleic Acids Res.

[27] Li B, Dewey CN (2011). RSEM: accurate transcript quantification from RNA-Seq data with or without a reference genome. BMC Bioinformatics.

[28] Robinson MD, McCarthy DJ, Smyth GK (2010). edgeR: a Bioconductor package for differential expression analysis of digital gene expression data. Bioinformatics.

[29] Love MI, Huber W, Anders S (2014). Moderated estimation of fold change and dispersion for RNA-seq data with DESeq2. Genome Biol.

[30] Anders S, McCarthy DJ, Chen Y, Okoniewski M, Smyth GK, Huber W, Robinson MD (2013). Count-based differential expression analysis of RNA sequencing data using R and Bioconductor. Nat Protoc.

[31] Love MI, Anders S, Kim V, Huber W (2015). RNA-Seq workflow: gene-level exploratory analysis and differential expression. F1000Research.

[32] Oliveros JC. VENNY. An interactive tool for comparing lists with Venn Diagrams. 2007–2015 http://bioinfogp.cnb.csic.es/tools/venny/index.html. Accessed Jan 2015 to Oct 2016.

[33] Pfaffl MW (2001). A new mathematical model for relative quantification in real-time RT–PCR. Nucleic Acids Res.

[34] Vandesompele J, De Preter K, Pattyn F, Poppe B, Van Roy N, De Paepe A, Speleman F (2002). Accurate normalization of real-time quantitative RT-PCR data by geometric averaging of multiple internal control genes. Genome Biol.

[35] Pfaffl MW, Tichopad A, Prgomet C, Neuvians TP (2004). Determination of stable housekeeping genes, differentially regulated target genes and sample integrity: BestKeeper–Excel-based tool using pair-wise correlations. Biotechnol Lett.

[36] Andersen CL, Jensen JL, Ørntoft TF (2004). Normalization of real-time quantitative reverse transcription-PCR data: a model-based variance estimation approach to identify genes suited for normalization, applied to bladder and colon cancer data sets. Cancer Res.

[37] Carr AC, Moore SD (2012). Robust quantification of polymerase chain reactions using global fitting. PLoS One.

[38] Ritz C, Spiess A-N (2008). qpcR: an R package for sigmoidal model selection in quantitative real-time polymerase chain reaction analysis. Bioinformatics.

[39] Kongtorp RT, Kjerstad A, Taksdal T, Guttvik A, Falk K (2004). Heart and skeletal muscle inflammation in Atlantic salmon, Salmo salar L.: a new infectious disease. J Fish Dis.

[40] Bruno D, Noguera PA, Poppe TT. A colour atlas of salmonid diseases, vol. 91. Springer Science & Business Media; 2013

[41] Stoltzfus CM, Shatkin AJ, Banerjee AK (1973). Absence of polyadenylic acid from reovirus messenger ribonucleic acid. J Biol Chem.

[42] Xu C, Evensen Ø, Munang’andu HM (2015). De novo assembly and transcriptome analysis of Atlantic salmon macrophage/dendritic-like TO cells following type I IFN treatment and Salmonid alphavirus subtype-3 infection. BMC Genomics.

[43] Moreton J, Dunham SP, Emes RD (2014). A consensus approach to vertebrate de novo transcriptome assembly from RNA-seq data: assembly of the duck (Anas platyrhynchos) transcriptome. Frontiers in Genetics.

[44] Liu S, Zhang Y, Zhou Z, Waldbieser G, Sun F, Lu J, Zhang J, Jiang Y, Zhang H, Wang X (2012). Efficient assembly and annotation of the transcriptome of catfish by RNA-Seq analysis of a doubled haploid homozygote. BMC Genomics.

[45] Wang S, Furmanek T, Kryvi H, Krossøy C, Totland GK, Grotmol S, Wargelius A (2014). Transcriptome sequencing of Atlantic salmon (Salmo salar L.) notochord prior to development of the vertebrae provides clues to regulation of positional fate, chordoblast lineage and mineralisation. BMC Genomics.

[46] Marancik D, Gao G, Paneru B, Ma H, Hernandez AG, Salem M, Yao J, Palti Y, Wiens GD (2014). Whole-body transcriptome of selectively bred, resistant-, control-, and susceptible-line rainbow trout following experimental challenge with Flavobacterium psychrophilum. Frontiers in Genetics.

[47] Davidson WS, Koop BF, Jones SJ, Iturra P, Vidal R, Maass A, Jonassen I, Lien S, Omholt SW (2010). Sequencing the genome of the Atlantic salmon (Salmo salar). Genome Biol.

[48] Hermansen RA, Hvidsten TR, Sandve SR, Liberles DA (2016). Extracting functional trends from whole genome duplication events using comparative genomics. Biological procedures online.

[49] Purcell MK, Kurath G, Garver KA, Herwig RP, Winton JR (2004). Quantitative expression profiling of immune response genes in rainbow trout following infectious haematopoietic necrosis virus (IHNV) infection or DNA vaccination. Fish Shellfish Immunol..

[50] Sagar S, Sharp F, Curran T (1988). Expression of c-fos protein in brain: metabolic mapping at the cellular level. Science.

[51] Shashoua VE (1991). Ependymin, a brain extracellular glycoprotein, and CNS plasticitya. Ann N Y Acad Sci.

[52] Sneddon LU, Schmidt R, Fang Y, Cossins AR (2011). Molecular correlates of social dominance: a novel role for ependymin in aggression. PLoS One.

[53] Suárez-Castillo EC, García-Arrarás JE (2007). Molecular evolution of the ependymin protein family: a necessary update. BMC Evol Biol.

[54] Tsoi S, Ewart K, Penny S, Melville K, Liebscher R, Brown L, Douglas S (2004). Identification of immune-relevant genes from Atlantic salmon using suppression subtractive hybridization. Marine Biotechnol.

[55] Volz DC, Hinton DE, Law JM, Kullman SW (2006). Dynamic gene expression changes precede dioxin-induced liver pathogenesis in medaka fish. Toxicol Sci.

[56] Brown T, Hori T, Ye C, Anderson DM, Rise M (2016). Functional genomic analysis of the impact of camelina (Camelina sativa) meal on Atlantic salmon (Salmo salar) distal intestine gene expression and physiology. Marine Biotechnol.

[57] Johansen L-H, Dahle MK, Wessel Ø, Timmerhaus G, Løvoll M, Røsæg M, Jørgensen SM, Rimstad E, Krasnov A (2016). Differences in gene expression in Atlantic salmon parr and smolt after challenge with Piscine orthoreovirus (PRV). Mol Immunol.

[58] Boehme KW, Lai CM, Dermody TS (2013). Mechanisms of reovirus bloodstream dissemination. Adv Virus Res.

[59] Clarke P, Tyler K (2003). Reovirus-induced apoptosis: a minireview. Apoptosis.

[60] Danthi P, Hansberger MW, Campbell JA, Forrest JC, Dermody TS (2006). JAM-A-independent, antibody-mediated uptake of reovirus into cells leads to apoptosis. J Virol.

[61] Doyle JD, Stencel-Baerenwald JE, Copeland CA, Rhoads JP, Brown JJ, Boyd KL, Atkinson JB, Dermody TS (2015). Diminished reovirus capsid stability alters disease pathogenesis and littermate transmission. PLoS Pathog.

[62] Irvin SC, Zurney J, Ooms LS, Chappell JD, Dermody TS, Sherry B (2012). A single-amino-acid polymorphism in reovirus protein μ2 determines repression of interferon signaling and modulates myocarditis. J Virol.

[63] Jeon Y, Lee JT (2011). YY1 tethers Xist RNA to the inactive X nucleation center. Cell.

[64] Nagano T, Mitchell JA, Sanz LA, Pauler FM, Ferguson-Smith AC, Feil R, Fraser P (2008). The air noncoding RNA epigenetically silences transcription by targeting G9a to chromatin. Science.

[65] Tripathi V, Ellis JD, Shen Z, Song DY, Pan Q, Watt AT, Freier SM, Bennett CF, Sharma A, Bubulya PA (2010). The nuclear-retained noncoding RNA MALAT1 regulates alternative splicing by modulating SR splicing factor phosphorylation. Mol Cell.

[66] Boltaña S, Valenzuela-Miranda D, Aguilar A, Mackenzie S, Gallardo-Escárate C. Long noncoding RNAs (lncRNAs) dynamics evidence immunomodulation during ISAV-Infected Atlantic salmon (Salmo salar). Sci Rep. 2016;6. 10.1038/srep22698 PMC4778034 26939752

[67] Corbett EL, Watt CJ, Walker N, Maher D, Williams BG, Raviglione MC, Dye C (2003). The growing burden of tuberculosis: global trends and interactions with the HIV epidemic. Arch Intern Med.

[68] Peltola V, Heikkinen T, Ruuskanen O, Jartti T, Hovi T, Kilpi T, Vainionpää R (2011). Temporal association between rhinovirus circulation in the community and invasive pneumococcal disease in children. Pediatr Infect Dis J.

[69] Shrestha S, Foxman B, Weinberger DM, Steiner C, Viboud C, Rohani P (2013). Identifying the interaction between influenza and pneumococcal pneumonia using incidence data. Sci Transl Med.

[70] Smith AM, Adler FR, Ribeiro RM, Gutenkunst RN, McAuley JL, McCullers JA, Perelson AS (2013). Kinetics of coinfection with influenza A virus and Streptococcus pneumoniae. PLoS Pathog.

[71] Weinberger DM, Klugman KP, Steiner CA, Simonsen L, Viboud C (2015). Association between respiratory syncytial virus activity and pneumococcal disease in infants: a time series analysis of US hospitalization data. PLoS Med.

[72] McNeilly F, Smyth J, Adair B, McNulty M. Synergism between chicken anemia virus (CAV) and avian reovirus following dual infection of 1-day-old chicks by a natural route. Avian Dis. 1995;532–537. 8561738

[73] Alonso M, Rodriguez S, Pérez-Prieto S (1999). Viral coinfection in salmonids: infectious pancreatic necrosis virus interferes with infectious hematopoietic necrosis virus. Arch Virol.

[74] LaPatra S, Lauda K, Jones G (1995). Aquareovirus interference mediated resistance to infectious hematopoietic necrosis virus. Vet Res.

[75] Kim HJ, Oseko N, Nishizawa T, Yoshimizu M (2009). Protection of rainbow trout from infectious hematopoietic necrosis (IHN) by injection of infectious pancreatic necrosis virus (IPNV) or Poly (I: C). Dis Aquat Organ.

[76] Johansen L-H, Krasnov A, Taksdal T, Moan I. Infectious pancreatic necrosis virus (IPNV) as risk factor in the development of heart and sceletal muscle inflammation (HSMI) in Atlantic salmon. In: Aquaculture Europe 2014. San Sebastian: European Aquaculture Society; 2014. https://www.was.org/easonline/AbstractDetail.aspx?i=3817.

