# Supplementary material for: De novo assembly of Sockeye salmon kidney transcriptomes reveal a limited early response to piscine reovirus with or without infectious hematopoietic necrosis virus superinfection
Source: BMC Genomics. 2016 Nov 2;17:848. doi: 10.1186/s12864-016-3196-y (PMC5094019; doi:10.1186/s12864-016-3196-y)
Supplement: Additional file 1: — Oligonucleotide primers and probes used in real time qPCR analyses. (PDF 153 kb) [file 12864_2016_3196_MOESM1_ESM.pdf]

Polinski *et al.* 2016 Additional file 1. Oligonucleotide primers and probes used in qPCR experiments.

Target	RNA-seq Component ID	Forward primer (5'->3')	Reverse primer (5' -> 3')	Probe (5' -> 3')
**PRV L1**	NA	TGCTAACACTCCAGGAGTCATTG	TGAATCCGCTGCAGATGAGTA	FAM-CGCCGGTAGCTCT-MGBNFQ
**IHNV N**	NA	AGAGCCAAGGCACTGTGCG	TTCTTTGCGGCTTGGTTGA	FAM-TGAGACTGAGCGGGACA-MGBNFQ
**B-actin**	c199144_g3	CAGGGAGAAGATGACCCAGA	GAGCGTAGCCCTCGTAGATG	
**EF1α**	c199620_g1_i9	CTTTGTGCCCATCTCTGGTT	GACGAAGGGGCTTATCTGTG	
**Dyn**	c186418_g5_i10	ACATCGAGAAAGACATCGCC	TCTCATGGGTCACGTAGCTG	
**ARP**	c196895_g3	GAAGGCTGTGGTTCTCATGG	GGTGAAGACAAAGCCCACAT	
**Neb**	c192081_g1	AGTCGGAGCCAAGAAGAACA	AATTCCTCAGGCCCTTCTGT	
**Epn**	c162029_g1	AATCACCACCTTTGGTCGAG	GGTCCAGAAGAAGTCGGATG	
**MMP-19**	c193589_g1	TCTTCACTGGGGTGCCCT	AGTAGCCCGTTCACACGC	
**HAL**	c188458_g3	TGCGTTGCTGTCCTCAGG	CACTGATGGCTGCCAGCT	
**Mx**	c201135_g2	GGTTGTGCCATGCAACGTT	GGCTTGGTCAGGATGCCTAAT	
**IL-1β**	c180606_g3	GGAGAGGTTAAAGGGTGGCGA	TGCCGACTCCAACTCCAACA	
**IL-8**	c190312_g1	CACTGAGATCATTGCCACTCTGA	ATGACCCTCTTGACCCACGG	
**CD2**	c199342_g10	TAAACCCCGTCTGACACACA	ATGGTCAGAGTAGCGCCTGT	
**Endod**	c200170_g3	GGGGTTATTCCCTACCTGGA	GGTCATTTTACCCCAGAGCA	
**Gimap**	c184181_g13	GTCCCACTGAGGCAAGTTGT	GGATTTCCCCAGGCAGTAAT	
**MAPK**	c193789_g3	ACAAGGCCCAATGACTGTTC	CCAAGCTGCTCCTGGTATTG	
**Picalm**	c201057_g6	CGTCTGAAGGAGATCGGTGT	CTGAGGTTGGGCATGCTATT	
**PIK3c2**	c202165_g1	AATTCCCAGACGTTGACAGG	TAGAGGCCGTCTCATCATCC	
**Pol**	c201714_g4	CTTCCCTGTCAAATGGAGGA	GTTTTGAACCGGTGCAGTTT	
**PItpn**	c199299_g2	CGCGTACGGCTCTACTAAGG	GAACTGACACTGGCTGGACA	
**Syngr**	c186878_g10	ACTGTTCATGGGAACCTTGG	AACCCCTACGTCTGCCAGTA	
