# Supplementary material for: De novo assembly of Sockeye salmon kidney transcriptomes reveal a limited early response to piscine reovirus with or without infectious hematopoietic necrosis virus superinfection
Source: BMC Genomics. 2016 Nov 2;17:848. doi: 10.1186/s12864-016-3196-y (PMC5094019; doi:10.1186/s12864-016-3196-y)
Supplement: Additional file 3: — Histopathological findings in Sockeye salmon exposed to Piscine orthoreovirus by injection and/or IHNV by cohabitation challenge. (PDF 262 kb) [file 12864_2016_3196_MOESM3_ESM.pdf]

Polinski *et al.* 2016 Additional file 3. Histopathological findings in Sockeye salmon exposed to Piscine orthoreovirus by injection and/or IHNV by cohabitation challenge.

	Mock PRV, mock IHNV				
**Fish ID**	**21 dpc PRV (7dpc IHNV)**		**Fish ID**	**62 dpc PRV (48dpc IHNV)**	
	**Kidney**	**Muscle**		**Kidney**	**Muscle**
**M928**	No pathological lesions found (NPLF).	Poor quality, scarce amounts of tissue. Possible exudation between fibers in red muscle	**M952**	Mid kidney. NPLF.	NPLF.
**929**	NPLF	Poor quality.	**953**	Mid kidney. NPLF.	NPLF.
**930**	NPLF	NPLF	**954**	Not tissue found.	Moderate degeneration of red muscle. Increased eosinophilia.
**931**	Mid kidney. NPLF.	No red muscle. Normal white muscle.	**955**	Mid kidney. NPLF.	Vacuolation of red muscle fibres.

	PRV+, mock IHNV				
**Fish ID**	**21 dpc PRV (7dpc IHNV)**		**Fish ID**	**62 dpc PRV (48 dpc IHNV)**	
	**Kidney**	**Muscle**		**Kidney**	**Muscle**
**M932**	Some bizarre lobed nuclei.	NPLF.	**M956**	NPLF.	Focal myositis in red muscle. (“longissimus dorsi”/dorsal carinal muscle)
**933**	NPLF	Some exudation between red and white muscle, possibly artifact.	**957**	NPLF.	No tissue found.
**934**	NPLF.	Small sample, not suitable for evaluation.	**958**	NPLF.	Focal myositis in red muscle. (“longissimus dorsi”/dorsal carinal muscle)
**935**	Not found	Slight to moderate degeneration of red muscle in one area.	**959**	NPLF.	Small piece of tissue.

	Mock PRV, IHNV challenged, but no IHNV detected in kidney				
**Fish ID**	**21 dpc PRV (7dpc IHNV)**		**Fish ID**	**62 dpc PRV (48 dpc IHNV)**	
	**Kidney**	**Muscle**		**Kidney**	**Muscle**
**194**	Head kidney with a few tubuli, with sparse eosinophilic material in their lumen (probably epithelial debris, regarded as normal). A focal moderately large bleeding, one small, sparse bleeding. Very few MMF.	Moderately large focus with degenerative changes in white muscle. A few red mucle fibers with clear, large vacuoles.	**265**	Mid kidney, with tubuli and glomeruli: Sparse –moderate amount of MMF and melaninfoci, like 216. Multifocally mild circulatory disturbances (blood interstitially and in vessels). Many tubuli with eosinphilic material in lumen, a few with small basophilic grains. Some tubuli with enlarged and/or non-similar epithelial nuclei/nuclei in different levels. Some PMN leucocytes interstitially.	One small focus of red muscle degenerative changes as in 211 and 213. One small focus with possible inflammation and lack of red muscle fibers close to epidermis.
**195**	Mid kidney, small parts of head kidney. Few MMF/melanin foci. Small amounts of eosinophilic material in lumen of some tubuli.	No tissue found.	**269**	Mostly mid kidney. Interstitium: Multifocally (some) -many PMN-leukocytes and mild circ. disturbances. Some MMF/melanin, but concentrated in sparse-moderate foci. Some eosinophilic material in lumen of tubuli.	Small area of large clear vacuoles in red muscle fibers. Degenerative changes like 211 and 213 in red muscle.
**197**	Head kidney with a few tubuli and a few foci of endocrine tissue. Very few MMF/few foci of melanin.	Epidermis and dermis is lacking (prob. sampling artefact). Focally possible exudation/oedema between some fibers of both red and white muscle. Mild degeneration, exudation and hypercellularity in red muscle.	**272**	Mid kidney. Few MMF/melanin foci. Small amounts of eosinophilic material in lumen of some tubuli.	Focus of red muscle with degenerative changes like in 211 and 213.
**203**	Head kidney, with some tubuli in 1/5 of the tissue. One foci of endocrine tissue. Few MMF/foci of melanin.	Mild degeneration in mid line red muscle (as in 199, 935 and others).	**276**	Interstitially sparse- (moderate) MMF/melanin and mild circ. disturbances. Some tubuli with nuclei like 265 (diff. levels etc).	Small amounts of red muscle. One small focus with loss of structure (mild form of degeneration).

	Mock PRV, IHNV challenged, IHNV in kidney				
**Fish ID**	**21 dpc PRV (7dpc IHNV)**		**Fish ID**	**62 dpc PRV (48 dpc IHNV)**	
	**Kidney**	**Muscle**		**Kidney**	**Muscle**
**193**	Head kidney with moderate foci with blood.	Most of epidermis and dermis is lacking (prob. artefact). Mod. large focus with degenerative changes in white muscle.	**267**	Mostly mid kidney. MMF/melanin like 265 (2) and 216 (more focally concentrated). Mild, multifocal circulatory disturbances intertitially. Similar to 265 (PMN, tubuli material).	A small area in dermis/red muscle with degenerative changes.
**199**	Head kidney. Multifocal/sonal areas with less cells, with pyknotic or karyorrhectic nuclei. Normal tissue between these areas. Few MMF/foci of melanin, but more concentrated/grouped than in 194 and 197.	Minor part of epidermis/dermis is missing (prob. preparation artefact). A few red muscle fibrs with large, clear vacuoles. Mild degeneration, exudation and hypercellularity in red muscle (mid line). Focally possible exudation/oedema between fibres of white muscle multifocally.	**275**	Mid kidney. Mild circ. disturbances interstitially, some MMF/sparse melanin. Eosinphilic material in lumen of some tubuli. A few PMN-leucocytes.	Focally some red muscle fibers with large, clear vacuoles. Melanin deposits basally for dermis. Moderate, focal area of red muscle basally to dermis with loss of structure (degenerative change).
**200**	Head kidney. Very limited amount of tissue. Few MMF/foci of melanin, one focus of endocrine tissue.	Parts of epidermis/dermis are lacking. Limited amount of red muscle, possible interfibrillar hypercellularity. Some of the red muscle fibres have large, clear vacuoles. Focally small foci of possible white muscle degeneration.			
**201**	Head and mid kidney. Small amounts of eosinophilic material in lumen of some tubuli. Some MMF/foci of melanin.	Moderate degeneration of mid line red muscle. Focally, small area of similar changes in white muscle close to edge and close to red mid line muscle. One small focus of possible sparse inflammation in torn red muscle subdermalt.			

	PRV+ ,<br>IHNV challenged,<br>but no IHNV detected<br>in kidney				
**Fish ID**	**21 dpc PRV (7dpc IHNV)**		**Fish ID**	**62 dpc PRV (48 dpc IHNV)**	
	**Kidney**	**Muscle**		**Kidney**	**Muscle**
**206**	Head kidney. Sp.-mod. tendency of congestion. Few MMF/foci of melanin.	Very small amount of red muscle, midline red muscle and large parts of epidermis/dermis is lacking (prob. artefact). Multifocally possible exudation/oedema between fibers of white muscle.	**277**	Mid kidney. Interstitially sparse-mod. MMF/melanin deposits, mild circ. disturbances, and some PMN-leucocytes interstitially. Some tubuli with nuclei like 265 (diff. levels etc.). Eosinphilic material in lumen of some tubuli.	Mid line red muscle with degenerative changes like in 211 and 213.
**209**	Head kidney. Some PMN-leucocytes in interstitium.	NPLF (no pathological lesions found).	**283**	Mid kidney. A bit crushed sample, difficult to interpret. Interstitially multifocal (sparse)- mod. MMF/ melanin (not single, but more focused), some PMN-leucocytes interstitially, possible very mild circ. disturbances. Some tubuli and –material as 265 and 277 + others.	Very small sample, no red muscle for evaluation, NPLF.
**211**	Head kidney. 6 patches of endocrine tissue. A few MMF/melanocytes.	Moderately large focus with some degenerative red muscle changes.	**285**	Mid kidney. Interstitially multifocally few MMF/melanin (distribution as in 269 And 283). Some tubuli with eosinophilic material in lumen, multifocally some with eosinophilic granula in tubuliepithelial cells. Focally mild circ. disturbances and a few PMN-leucoccytes in the interstitium. Few tubuli with nuclei like 265 (diff. levels etc).	Small sample, small part only with epidermis, dermis and red muscle: A few red muscle fibers with large, clear vacuoles.
**213**	Head kidney. A few MMF/melaninfoci. 2 tubuli and 1 focus of endocrine tissue.	Some minor vacuolization of a few red m. fibres. Focally degenerative red muscle changes similar to 211 and 213.	**288**	Mid kidney. Interstitially few -some MMF/melanindeposits (distribution as in 269 and 283 + others) mild circ. disturbances, some – many PMN-leucocytes interstitially. Some tubuli with eosinophilic material in lumen. Few tubuli with nuclei like 265 (diff. levels etc). Relatively high proportion of blood cells in vessels consists of leucocytes, could be post-mortem artefact.	Relatively small sample. Several red muscle fibers with large, clear vacuoles.

	PRV+ , IHNV challenged, with IHNV in kidney				
**Fish ID**	**21 dpc PRV (7dpc IHNV)**		**Fish ID**	**62 dpc PRV (48 dpc IHNV)**	
	**Kidney**	**Muscle**		**Kidney**	**Muscle**
**207**	Head kidney. Three moderately large foci of endocrine tissue. Some MMF/foci of melanin. A small - moderately large focus with few possible necrotic cells.	Very small amount of red muscle, focally possible interfibrillar hypercellularity.	**282**	Mid kidney. Interstitially moderate+ to focally severe circ. disturbances. Few MMF/ small melanin deposits, very mild erytrophagocytosis, some PMN-leucocytes. Tubuli and –material in their lumen like 265 and 277 + others.	Some melanin subdermal. Multifocally a few white muscle fibers with degenerative centres, but without inflammatory cell infiltration.
**210**	Mainly head kidney, but about 1/6 of the sample contains tubuli. Sparse-moderate circ. disturbances, incl. to small, possible bleedings. A few MMF/melanin deposits.	Moderate degenerative changes in red muscle (like 211, 213). One small foci of myositis in the degenerated red muscle, does not resemble the inflammation seen in HSMI. A few possible degenerated white muscle fibers, close to dermis/edge of white muscle.			
**214**	Head kidney, one focus of endocrine tissue. Blood in larger vessels and easily observed/"standing out " sinusoids: Possible mild circulatory disturbances. A few MMF/melaninfoci.	One small inflammatory focus and a vessel with sparse perivascular inflammatory cell infiltration and sparse oedema in white muscle. One small area of possible degeneration and inflammation of red muscle, degenerative changes similar to changes in 211 and 213.			
**216**	Head kidney, small piece: A few tubuli, a few MMF/melanin foci. Some areas w. sp.-mod. MMF/melanin foci intersititially, between tubuli. Small amounts of eosinophilic material in tubuli, probably epithelial debris. Relatively high proportion of PMN leucocytes interstitially (personal impression). Small pieces, a lot of possibilities for «edge artefacts», difficult to interpret.	A minor focus of inflammation in white muscle. Focal degenerative changes of red muscle red muscle, like in 211 and 213.			
