# Supplementary material for: De novo assembly of Sockeye salmon kidney transcriptomes reveal a limited early response to piscine reovirus with or without infectious hematopoietic necrosis virus superinfection
Source: BMC Genomics. 2016 Nov 2;17:848. doi: 10.1186/s12864-016-3196-y (PMC5094019; doi:10.1186/s12864-016-3196-y)

Polinski *et al.* 2016 Additional file 8. Putative DEGs identified by preliminary DESeq and edgeR analysis and subsequent qPCR validation

1) DEGs identified by preliminary DESeq (prior version to DESeq2) and edgR analysis. Unigenes identified by both edgeR and DESeq from unfiltered RSEM count data found to be differentially expressed ($p<0.05$) in response to PRV. For each unigene, the Log_2 fold change and 0.05 FDR adjusted p-value are presented as calculated by either edgeR or DESeq software packages. Best UniProt annotation (blastx E value < 1e-5) is provided where available.

Analysis	Unigene ID	Log_2FC	DESeq p value	edgeR p value	blastx annotation
**Group 1 vs 2 (14d mock vs PRV)**					
	c184181_g13	-9.538	0.0363	0.0015	GTPase IMAP family member
	c202165_g1	1.469	0.0199	0.0058	Phosphatidylinositol 4-phosphate 3-kinase subunit
	c193180_g3	1.52	0.05	0.0490	
	c190077_g2	1.585	0.0363	0.0396	
	c185795_g1	1.625	0.0245	0.0325	
	c185722_g5	1.629	0.0477	0.0265	
	c200076_g3	1.967	0.0363	0.0124	
	c177541_g1	2.011	0.0363	0.0473	
	c190986_g6	2.043	0.0199	0.0291	
	c178668_g1	2.284	0.0439	0.0066	
	c148788_g1	2.322	0.0363	0.0188	
	c192081_g1	2.334	0.0134	0.0006	Nebulin
	c198428_g5	2.735	0.0245	0.0124	
	c186377_g2	3.197	0.0363	0.0124	
**Group 3 vs 6 (21d mock vs PRV)**					
	c199342_g10	-11.145	0.0000	0.0000	T-cell surface antigen CD2
	c115940_g1	-9.721	0.0004	0.0000	
	c90613_g1	-9.547	0.0002	0.0000	
	c188778_g5	-8.921	0.0000	0.0000	
	c172905_g1	-8.686	0.0011	0.0000	
	c199342_g11	-8.213	0.0035	0.0001	
	c180882_g2	-8.21	0.0000	0.0000	Neuropeptide FF receptor 1
	c197743_g10	-8.028	0.0047	0.0001	
	c117977_g1	-7.163	0.0160	0.0008	
	c169198_g1	-7.089	0.0000	0.0000	
	c160546_g1	-6.543	0.0250	0.0017	
	c192145_g1	-4.479	0.0000	0.0000	
	c199348_g2	-4.04	0.0000	0.0330	
	c192145_g2	-3.693	0.0005	0.0000	
	c5326_g1	-3.378	0.0001	0.0220	
	c160845_g1	-3.373	0.0000	0.0270	Y+L amino acid transporter 1
	c202547_g2	-2.439	0.0210	0.0049	Chromobox protein homolog 2
	c195952_g8	-2.237	0.0037	0.0270	
	c190607_g9	-2.227	0.0260	0.0110	
	c199005_g2	-2.161	0.0470	0.0100	
	c199829_g2	-2.158	0.0001	0.0100	

c201595_g1	-2.141	0.0004	0.0037	
c198581_g1	-2.087	0.0050	0.0240	
c272614_g1	-2.028	0.0430	0.0240	
c254542_g1	-2.004	0.0250	0.0240	Nucleoside diphosphate kinase, mitochondrial
c196929_g5	-1.953	0.0320	0.0160	
c196227_g3	-1.834	0.0001	0.0190	Fibrillin-1
c184857_g4	-1.806	0.0002	0.0340	
c175785_g1	-1.659	0.0200	0.0330	RAS guanyl-releasing protein 4
c202147_g2	-1.516	0.0064	0.0370	Filamin-B
c199191_g7	-1.503	0.0009	0.0240	Collagen alpha-1(XII) chain
c189708_g1	-1.492	0.0001	0.0240	Talin-1
c182450_g1	-1.486	0.0007	0.0290	Beta-1,3-N-acetylglucosaminyltransferase 2
c201057_g6	-1.485	0.0038	0.0370	Phosphatidylinositol-binding clathrin assembly protein
c191266_g1	-1.478	0.0002	0.0240	Collagen alpha-1(I) chain
c194168_g1	-1.44	0.0000	0.0110	Cytoplasmic dynein 1 heavy chain 1
c196282_g5	-1.372	0.0001	0.0230	Max dimerization protein 4
c183288_g2	-1.371	0.0110	0.0330	Phosphatidate cytidylyltransferase 1
c191519_g6	-1.366	0.0470	0.0440	Monocarboxylate transporter 4
c193789_g3	-1.362	0.0064	0.0140	Mitogen-activated protein kinase kinase 5
c168659_g1	-1.349	0.0039	0.0220	Ubiquitin-associated SH3 domain-containing prot B
c186878_g10	-1.325	0.0009	0.0200	Synaptogyrin-1
c194965_g2	-1.325	0.0130	0.0320	Membrane-assoc phosphatidylinositol transfer prot 2
c173530_g1	-1.25	0.0008	0.0410	WD repeat-containing protein 81
c201714_g4	-1.206	0.0110	0.0490	Transposon Ty3-I Gag-Pol polyprotein
c187171_g2	-1.165	0.0240	0.0430	
c191748_g2	-1.163	0.0015	0.0230	Beta-galactoside alpha-2,6-sialyltransferase 2
c193053_g4	-1.159	0.0140	0.0410	
c199299_g2	-1.123	0.0086	0.0430	Membrane-assoc phosphatidylinositol transfer prot 2
c198536_g5	1.194	0.0003	0.0017	
c198536_g2	1.208	0.0003	0.0022	
c195388_g2	1.237	0.0068	0.0230	
c73329_g1	1.407	0.0008	0.0049	
c196486_g1	1.414	0.0370	0.0360	Zinc finger protein 541
c158690_g2	1.424	0.0017	0.0110	
c195556_g4	1.582	0.0000	0.0001	
c198385_g3	1.598	0.0310	0.0450	
c199128_g2	1.63	0.0300	0.0420	
c200170_g3	1.649	0.0003	0.0000	Endonuclease domain-containing protein
c201290_g2	1.668	0.0000	0.0000	
c192433_g4	1.82	0.0006	0.0019	
c199128_g1	1.869	0.0000	0.0026	
c190786_g1	1.871	0.0010	0.0078	
c162029_g1	3.011	0.0000	0.0000	Ependymin
c139187_g2	3.146	0.0039	0.0480	
c180425_g1	3.564	0.0110	0.0015	
c198881_g4	7.871	0.0062	0.0002	

2) qPCR calculated expression of select genes in response to PRV. Unigenses identified as differentially expressed in response to PRV at either 14 dpc or 21 dpc by preliminary RNA-seq analysis using DESeq and edgeR ($p<0.05$), but not validated by analysis using DESeq2. Additional time points (2 dpc, 5 dpc) were included in the analysis to better understand the temporal significance of PRV on transcription. Normalized relative quantities for each transcript were calculated using the ViiA™ 7 Software v1.2 (Applied Biosystems) for Comparative CT (ΔΔCT), in reference to *EF1α* and *arp* which showed stable expression. At each time point, significant differential expression between mock and PRV groups is indicated at $p<0.05$ (*) by t-test using Graphpad Prism 5.0. With regard to Cluster of differentiation 2 (C*D2*), quantities for non-expression are presented as zero in reference to 40 amplification cycles which precluded quantitative statistical comparison.

A. CD2 (c199342_g10)

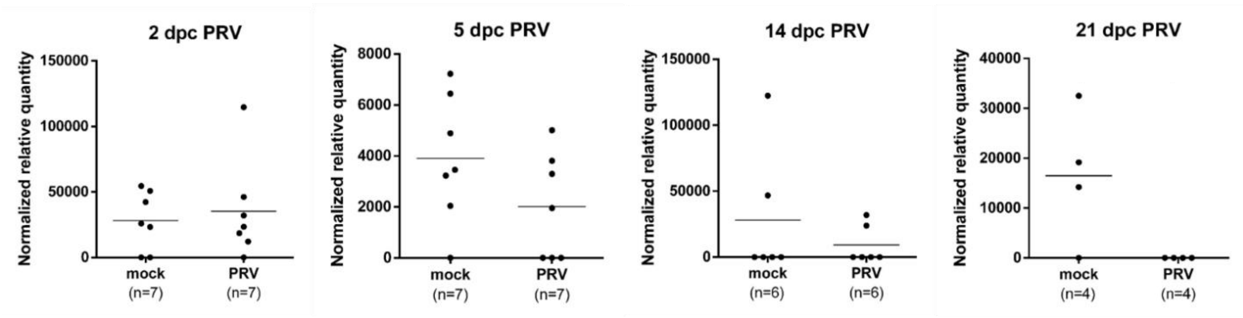

B. Endonuclease domain-containing protein (c200170_g3)

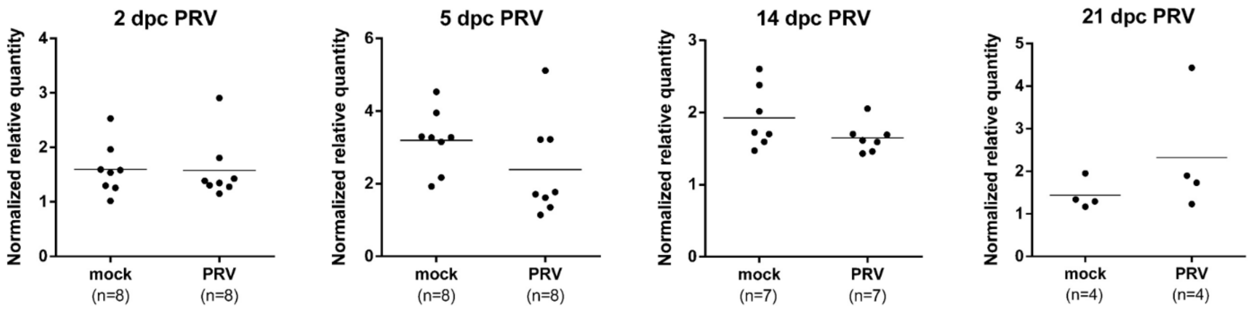

C. GTPase IMAP family member (c184181_g13)

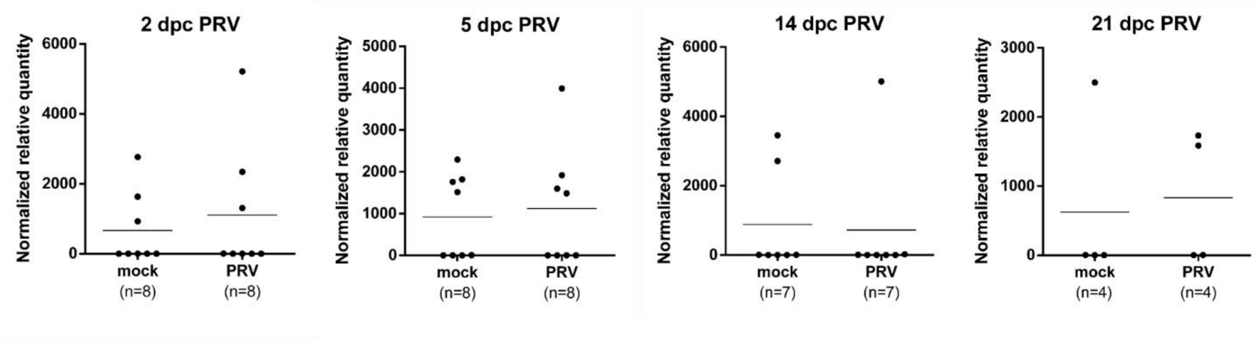

D. MAP Kinase 5 (c193789_g3)

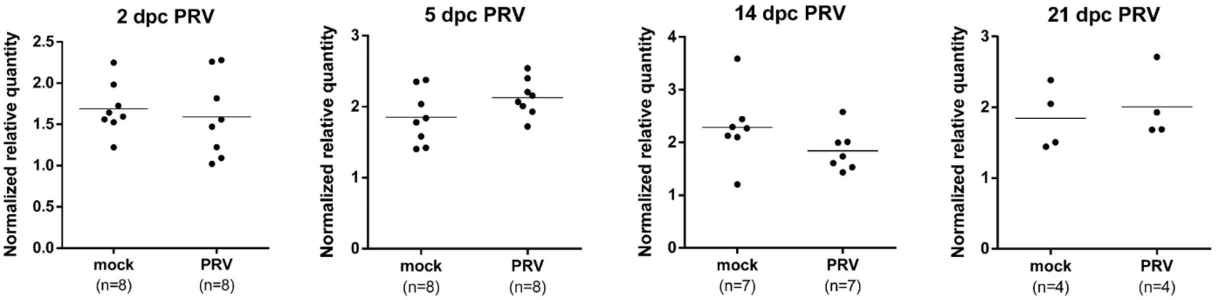

E. Phosphatidylinositol binding protein (c201057_g6)

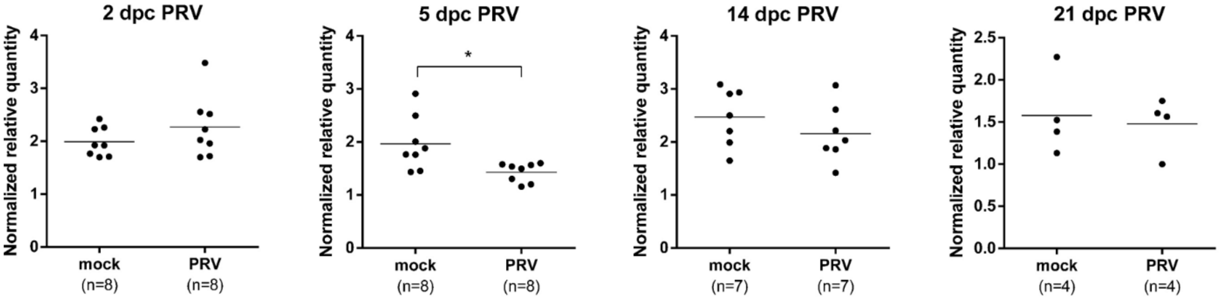

F. Phosphatidylinositol 3-kinase domain c2 (c202165_g1)

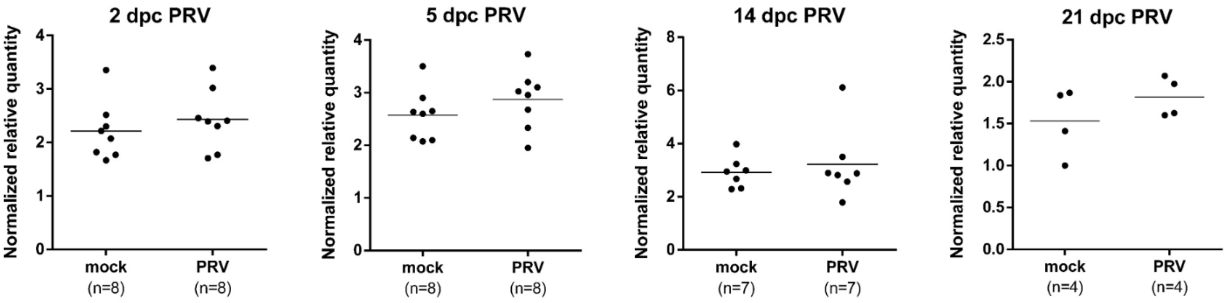

G. Pol polyprotein (c201714_g4)

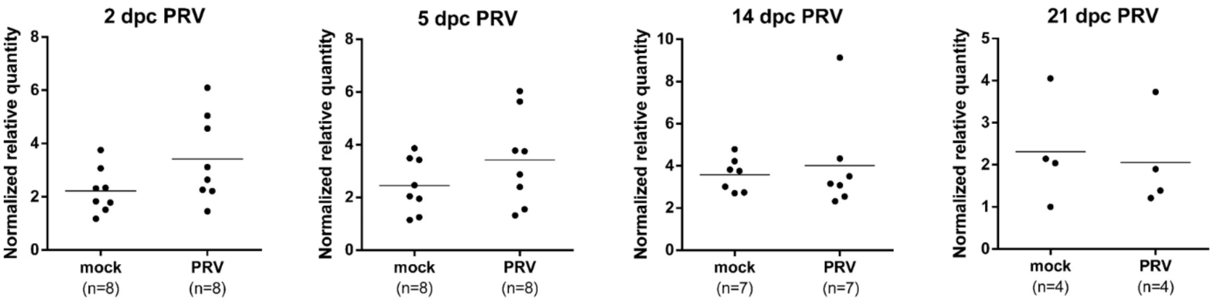

H. Phosphatidylinositol transfer protein (c199299_g2)

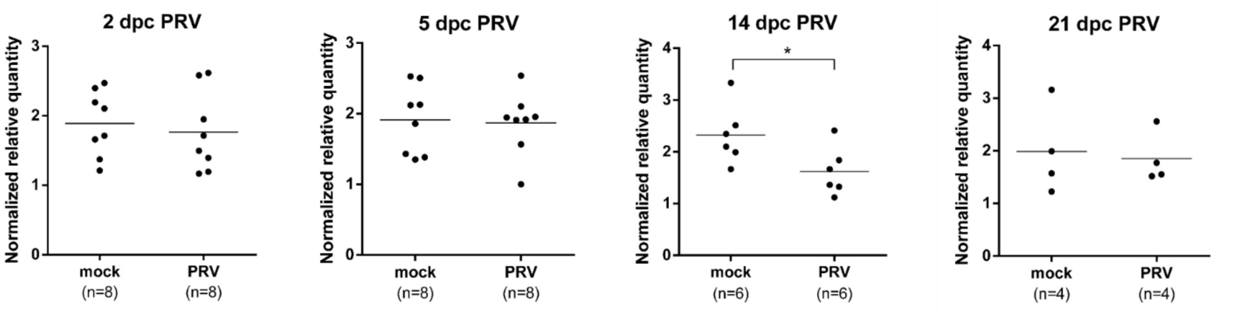

I. Synaptogyrin (c186878_g10)

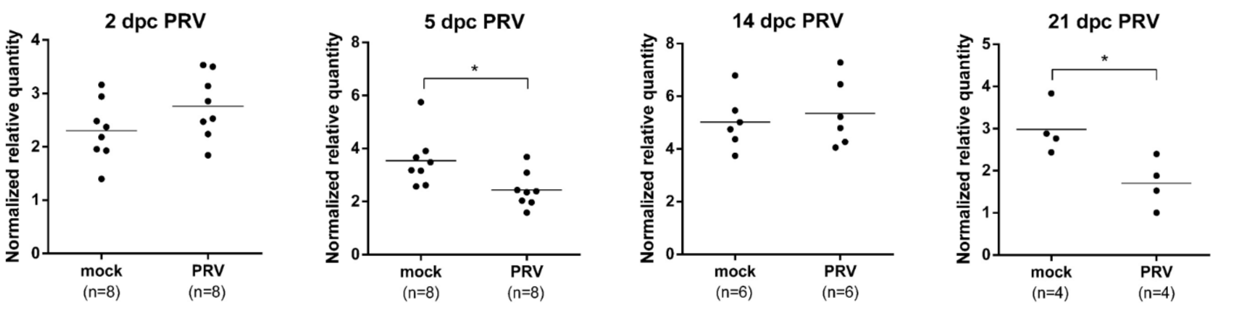

Supplement: Additional file 8: — Putative DEGs identified by preliminary DESeq and edgeR analysis and subsequent qPCR validation. (PDF 1454 kb) [file 12864_2016_3196_MOESM8_ESM.pdf]
